# The genome of common long-arm octopus *Octopus minor*

**DOI:** 10.1093/gigascience/giy119

**Published:** 2018-09-25

**Authors:** Bo-Mi Kim, Seunghyun Kang, Do-Hwan Ahn, Seung-Hyun Jung, Hwanseok Rhee, Jong Su Yoo, Jong-Eun Lee, SeungJae Lee, Yong-Hee Han, Kyoung-Bin Ryu, Sung-Jin Cho, Hyun Park, Hye Suck An

**Affiliations:** 1 Unit of Polar Genomics, Korea Polar Research Institute (KOPRI), Incheon 21990, Korea; 2 Department of Genetic Resources Research, National Marine Biodiversity Institute of Korea (MABIK), Janghang-eup, Seochun-gun, Chungchungnam-do 33662, Korea; 3 Genomics Lab, Cluster Center, DNA Link, Inc., 150, Bugahyeon-ro, Seodaemun-gu, Seoul 03759, Korea.1; 4 School of Biological Sciences, College of Natural Sciences, Chungbuk National University, Cheongju, Chungbuk 28644, Korea; 5 Polar Sciences, University of Science & Technology, Yuseong-gu, Daejeon 34113, Korea

**Keywords:** octopus genome, cephalopods, adaptation and evolution, long-read sequencing

## Abstract

**Background:**

The common long-arm octopus (*Octopus minor*) is found in mudflats of subtidal zones and faces numerous environmental challenges. The ability to adapt its morphology and behavioral repertoire to diverse environmental conditions makes the species a promising model for understanding genomic adaptation and evolution in cephalopods.

**Findings:**

The final genome assembly of *O. minor* is 5.09 Gb, with a contig N50 size of 197 kb and longest size of 3.027 Mb, from a total of 419 Gb raw reads generated using the Pacific Biosciences RS II platform. We identified 30,010 genes; 44.43% of the genome is composed of repeat elements. The genome-wide phylogenetic tree indicated the divergence time between *O. minor* and *Octopus bimaculoides* was estimated to be 43 million years ago based on single-copy orthologous genes. In total, 178 gene families are expanded in *O. minor* in the 14 bilaterian species.

**Conclusions:**

We found that the *O. minor* genome was larger than that of closely related *O. bimaculoides*, and this difference could be explained by enlarged introns and recently diversified transposable elements. The high-quality *O. minor* genome assembly provides a valuable resource for understanding octopus genome evolution and the molecular basis of adaptations to mudflats.

## Background

Cephalopods (e.g., cuttlefish, nautilus, octopus, and squid) belong to the phylum Mollusca, which is one of the most diverse phylum within Lophotrochozoa. Regardless of their evolutionary, biological, and economic significance, their genome information is still limited to a few species [1–4].

Cephalopods have interesting biological characteristics such as an extraordinary life-history plasticity, rapid growth, short life span, large brain, and sophisticated sense organs with a complex nervous system [5]. The ability to adapt their morphology and behavioral repertoire to diverse environmental conditions and their capacity for learning and memory are common traits in cephalopods but have rarely been observed in other invertebrates [6]. Many cephalopod species have been considered for fisheries and are promising candidates for aquaculture. There are an estimated 1,000 cephalopod species (∼700 known marine-living species), and octopods are among the most well-known representatives of the class, including more than 150 species worldwide [7]. Studies have evaluated the biological machinery underlying the fundamental nervous system functions, strong behavioral plasticity, and learning ability in octopods [8, 9].

**Figure 1:**
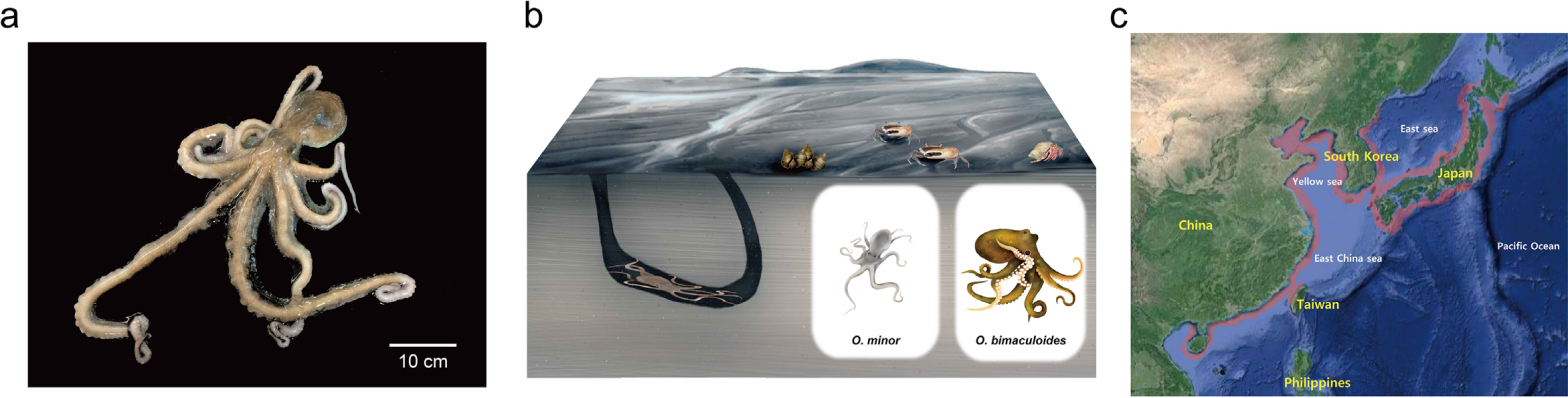
Common long-arm octopus (*Octopus minor*). **(a)** Photograph of *O. minor*. (**b)** Habitat structure of mudflats and phenotypic differences between *O. minor* and *Octopusbimaculoides. Octopusminor* has a smaller body size and possesses longer, thinner arms than those of *O. bimaculoides*. **(c)** The distribution of *O. minor* is shown in red. The distribution map was updated from Roper et al. (1984)^11^.

*Octopus minor* (Sasaki, 1920) (NCBI taxon ID:515824), also known as the common long-arm octopus, is a benthic littoral species and a major commercial fishery product with a high annual yield [10]. *Octopusminor* is relatively small and possesses a shorter life cycle (approximately 1 year), thinner arms, and a lower ratio between head size and arm length compared to those of other *Octopus* species (Fig. 1a and b). The species is widely distributed in northeast Asia, particularly in coastal regions of South Korea, China, and Japan (Fig. 1c). Most *O. minor* habitats are mud and mud-sand in well-developed mudflats of coastal regions; they spawn in holes on the mudflat by digging with the whole body. Thus, they are subjected to the harsh environmental conditions of mudflats, including diurnal temperature changes, steep salinity and pH gradients, desiccation, wave action and tides, oxygen availability, and interrupted feeding. Owing to the ability of *O. minor* to tolerate environmental fluctuations, it is a promising organism for studies of the molecular basis of plasticity and mechanisms underlying adaptation to harsh environmental conditions, although relevant information is scarce. To make full use of this emerging cephalopod model system and to understand the interesting features of *O. minor*, including its plasticity in mudflats and genetic evolution, a high-quality reference genome is required.

The published genome and multiple transcriptomes of the California two-spot octopus *Octopus bimaculoides* have provided valuable information on genomic traits (e.g., gene family expansion, genome rearrangements, and transposable element activity) related to the evolution of neural complexity and morphological innovations [3]. In this study, we report a high-quality genome assembly and annotation for *O. minor*. We compare the genomes of *O. minor* and *O. bimaculoides* and provide evidence that the expansion of genes and/or gene families is related to adaptation to the harsh environmental conditions of mudflats.

## Data Description

### Genome sequencing and annotation

*Octopusminor* genomic DNA was extracted from leg muscle tissues. The average coverage of Single-Molecular Real-Time (SMRT) sequences was ∼76-fold using P6-C4 sequence chemistry from genomic DNA libraries that was sequenced using the Pacific Biosciences (PacBio) RS II platform. The average subread length was 9.2 kb (Supplementary Table S1). For genome size estimation, *k*-mer analysis was performed using Jellyfish ver. 2.1.3 (Jellyfish, RRID:SCR_005491) [12] with paired-end sequences of the genomic DNA libraries. The *O. minor* genome was estimated to be 5.1 Gb (Supplementary Figs. S1 and S2). The *de novo* assembly generated using FALCON-Unzip assembler ver. 0.4 was 5.09 Gb with 41,584 contigs (Falcon, RRID:SCR_016089) [13]. Finally, evaluation of the genome completeness was checked using Benchmarking Universal Single-Copy Orthologs (BUSCO) ver. 1.22 (BUSCO, RRID:SCR_015008) [14] (Table 1).

**Table 1:** BUSCO evaluated for the completeness of the *O. minor* genome assembly

	Eukaryote		Metazoa	
	Count	%	Count	%
Complete BUSCOs (C)	224	73.9	745	76.2
Complete and single-copy BUSCOs (S)	193	63.7	628	64.2
Complete and duplicated BUSCOs (D)	31	10.2	117	12
Fragmented BUSCOs (F)	26	8.6	82	8.4
Missing BUSCOs (M)	53	17.5	151	15.4
Total BUSCO groups searched	303		978	

Total RNA was extracted from 13 tissues (brain, branchial heart, buccal mass, eye, heart, kidney, liver, ovary, poison gland, siphon, skin, and suckers) using the RNeasy Mini Kit (Qiagen, Hilden, Germany) according to the manufacturer's instructions. RNA quality was confirmed using an Agilent Bioanalyzer. Isoform sequencing was performed using pooled RNA from 13 organs. Library construction and sequencing were performed using PacBio RS II (Supplementary Table S2). The SMRTbell library for Iso-seq was sequenced using 16 SMRT cells (1–2 kb, three cells; 2–3 kb, six cells; and 3–6 kb, seven cells). Reads were identified using the SMRT Analysis ver. 2.3 RS_IsoSeq.1 classification protocol. All full-length reads derived from the same isoform were clustered, and consensus sequences were polished using the TOFU pipeline (isoseq-tofu) [15]. Additionally, chimeras of consensus sequences generated during experiments and TOFU pipeline were removed using in-house script.

MAKER ver. 2.28 was used for genome annotation (MAKER, RRID:SCR_005309) [16]. First, repetitive elements were identified using RepeatMasker ver. 4.0.7 (RepeatMasker, RRID:SCR_012954) [17]. A *de novo* repeat library was constructed using RepeatModeler ver. 1.0.3 (RepeatModeler, RRID:SCR_015027) [18], including RECON ver. 1.08 [19] and RepeatScout ver. 1.0.5 (RepeatScout, RRID:SCR_014653) [20], with default parameters. Consensus sequences and classification information for each repeat family were generated, and tandem repeats, including simple repeats, satellites, and low-complexity repeats, were predicted using Tandem Repeats Finder [15]. This masked genome sequence was used for *ab initio* gene prediction with SNAP software (SNAP—SNP Annotation and Proxy Search, RRID:SCR_002127) [21]. Subsequently, alignments of expressed sequence tags with the Basic Local Alignment Search Tool n ver. 2.2.28+ (BLASTN, RRID:SCR_001598) and protein information from tBLASTx ver. 2.2.28+ (TBLASTX, RRID:SCR_011823) were included. The *de novo* repeat library of *O. minor* from RepeatModeler was used for RepeatMasker. Proteins from sequenced molluscs (*Lottia gigantea, Crassostrea gigas*, and *Aplysia californica*) and an octopus species (*O. bimaculoides*) were included in the analysis. Transcriptome assembly results were used for expressed sequence tags. Next, MAKER polished the alignments using Exonerate, which provided integrated information for SNAP annotation. Using MAKER, the final gene model was selected and revised considering all information. A total of 30,010 *O. minor* genes were predicted using MAKER. The Infernal software package ver. 1.1 (Infernal, RRID:SCR_011809) [22] and covariance models from the Rfam (Rfam, RRID:SCR_007891) [23] database were used to identify other noncoding RNAs in the *O. minor* scaffold. Putative tRNA genes were identified using tRNAscan-SE ver. 1.4 (tRNAscan-SE, RRID:SCR_010835) [24]. tRNAscan-SE uses a covariance model that scores candidates based on their sequence and predicted secondary structures.

The mean size of *O. minor* genes was 23.6 kb, with an average intron length of 5.4 kb (4.2 introns per gene) (Supplementary Table S3). The *O. minor* genome contained 30,010 protein-coding genes (Table 2), of which 96% were annotated based on known proteins in public databases, and 79% were similar to *O. bimaculoides* genes (Supplementary Table S4).

**Table 2:** Overview of the assembly and annotation of the *Octopus minor* genome

Total length (bp)	5,090,349,614
Number of contigs	41,584
Contig N50 (bp)	196,941
Largest contigs (bp)	3,027,443
GC (Guanine-Cytosine) content (%)	36.33
Number of protein-coding genes	30,010

### Comparative genomic analyses and duplicate genes

To resolve gene family evolution in the *O. minor* genome, we classified orthologous gene clusters (Supplementary Table S5) from 14 species and found evidence for the recent expansion of low-copy gene duplicates and the expansion of large gene families. Orthologous groups were identified using both OrthoMCL ver. 2.0.9 [25] and Pfam (Pfam, RRID:SCR_004726) [26] domain assignments. OrthoMCL generated a graphical representation of sequence relationships, which was then divided into subgraphs using the Markov clustering algorithm from multiple eukaryotic genomes [25]. The default parameters and options of OrthoMCL were used for all steps, together with the genomes of 14 species (Supplementary Table S5). For *O. minor*, the coding sequence from the MAKER annotation pipeline was used. To construct a phylogenetic tree and estimate the divergence time, 202 one:one single-copy orthologous genes were used. Using the Probabilistic Alignment Kit ver.140603 [27], protein-coding genes were aligned with the codon alignment option, and poorly aligned regions with gaps were eliminated using Gblocks ver. 0.91b [28] with a codon model. A maximum-likelihood tree was built using RAxML ver. 8.2.4 (RAxML, RRID:SCR_006086) [29] with 1,000 bootstrap replicates, and the divergence time was calibrated using TimeTree [30]. The average gene gain-loss was identified using CAFE ver. 4.0 [31] with *P* value < 0.05.

Sequence divergence was estimated by calculating *d*_S_ values using the yn00 program from the PAML package ver. 4.7a (PAML, RRID:SCR_014932) [32]. The Jukes–Cantor distances were adjusted using the Jukes–Cantor formula *d*_XY_ = -(3/4)ln(1–4/3D), where D is the proportion of nucleotide differences between the sequences. The time estimation was calibrated by assuming *d*_S_ of ∼1 is 135 million years [7].

Gene family analyses of specific genes of interest were manually curated using manual gene search methods. Gene or gene family targets identified in the genomes of *O. bimaculoides, C. gigas, L. gigantea, Capitella teleta*, and *Homo sapiens* were directly mapped to the *O. minor* genome database by a local BLAST analysis. Alignments were generated using Clustal Omega ver. 1.2.4 [33] and Multiple Sequence Comparison by Log-Expectation ver. 3.8.31 (MUSCLE, RRID:SCR_011812) [34], and phylogenetic trees were built using FastTree [35] or RAxML with 1,000 bootstrap replicates.

**Figure 2:**
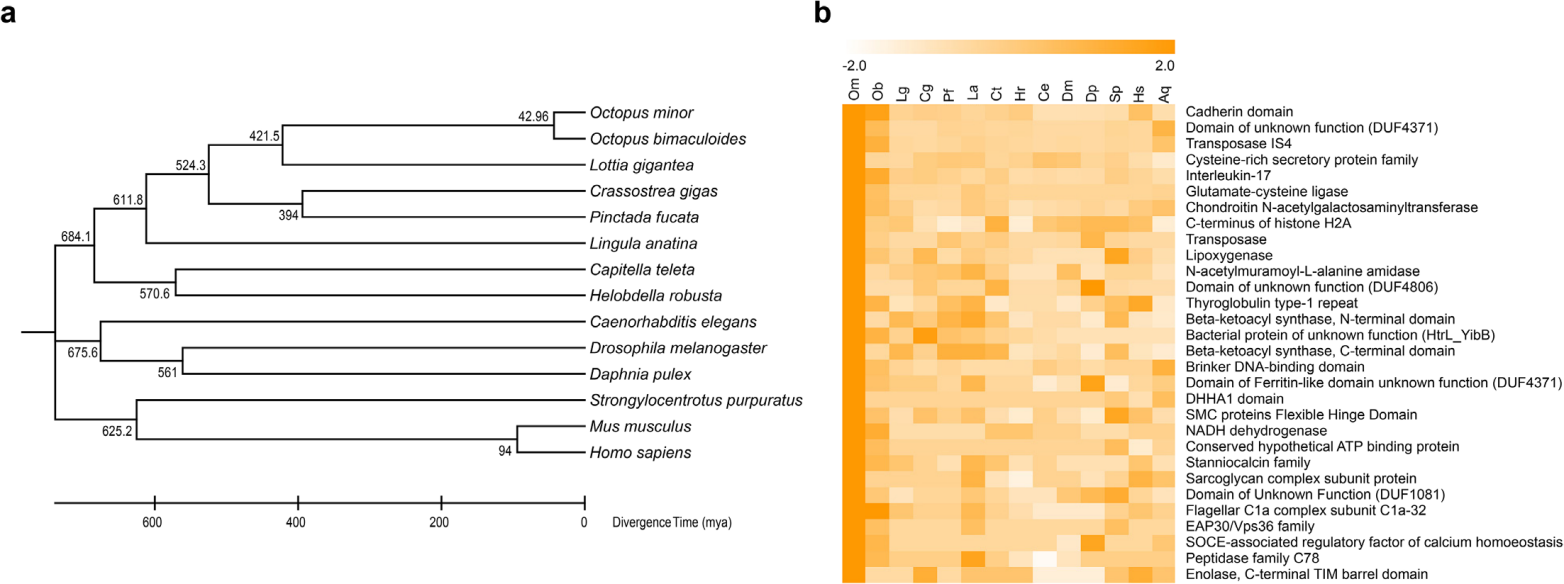
Gene family analysis for 14 bilaterian species. **(a)** Divergence times estimated from genome sequences of 14 bilaterian species. **(b)** Heat map of expanded Pfam domains in the *O. minor* genome. OM, *Octopus minor*; OB, *Octopus bimaculoides*; LG, *Lottia gigantea*; CG, *Crassostrea gigas*; PF, *Pinctada fucata*; LA, *Lingula anatina*; CT, *Capitella teleta*; HR, *Helobdella robusta*; CE, *Caenorhabditis elegans*; DM, *Drosophila melanogaster*; DP, *Daphnia pulex*; SP, *Strongylocentrotus purpuratus*; MM, *Mus musculus*; HS, *Homo sapiens*.

Gene gain-loss analysis indicated significantly greater gene family expansion in *O. minor* (178 gene families) compared to other species, e.g., interleukin-17, G protein-coupled receptor proteins, zinc-finger of C2H2 type, heat shock protein (HSP) 70 proteins, right and cadherin-like domains (Supplementary Tables S6–S8). The divergence time between *O. minor* and *O. bimaculoides* was estimated to be 43 million years ago (Mya) based on single-copy orthologous genes (Fig. 2a) Further, Pfam domain and EggNOG metazoan database searches consistently showed the expansion of gene families, including the cadherin and protocadherin domains and interleukin-17 (Fig. 2b, Supplementary Tables S9 and S10).

Previously, 168 protocadherin (*pcdhs*) genes were annotated in the genome of *O. bimaculoides*, which is the largest number among sequenced metazoan genomes [3]. In the case of the C2H2 zinc-finger gene family, approximately 1,800 C2H2 genes were annotated in the *O. bimaculoides* genome. The drastic expansions were also observed in the genome of *O. minor*, as 303 and 2,289 genes were annotated for *pcdhs* and the C2H2 zinc-finger gene family, respectively. We assume that the expansion at

patterns are unique to the genus *Octopus*, as the expansion pattern was not detected in squid and the *pcdhs* seem to have expanded after octopuses diverged from squid (≈135 Mya) [3]. Since we estimated that *O. minor* diverged from the genus *Octopus*, the extraordinary expansions of both gene families are presumably *Octopus* specific.

### Transposable element annotation and expansions

The *O. minor* genome (5.1 Gb) is composed of 44% repetitive sequences and 0.68% coding sequences, while the *O. bimaculoides* genome (2.7 Gb) made up of 35% repetitive sequences and 1.08% coding sequences. Repeats were dominated by simple repeats (14.7% of genome) and transposable elements (TEs), especially DNA transposons and long interspersed elements (LINEs), which were more abundant in the *O. minor* genome than in the *O. bimaculoides* genome (Supplementary Tables S11–S13). In an analysis of genes (i.e., exons and introns) and intergenic sequences, TEs were highly distributed in the intergenic sequence regions in both species (Supplementary Fig. S4). In particular, TE accumulation in intergenic sequence regions was significantly greater in *O. minor* than in O. *bimaculoides*. The larger number of gene size and higher repeat content may explain the larger genome of *O. minor* compared with *O. bimaculoides*.

**Figure 3:**
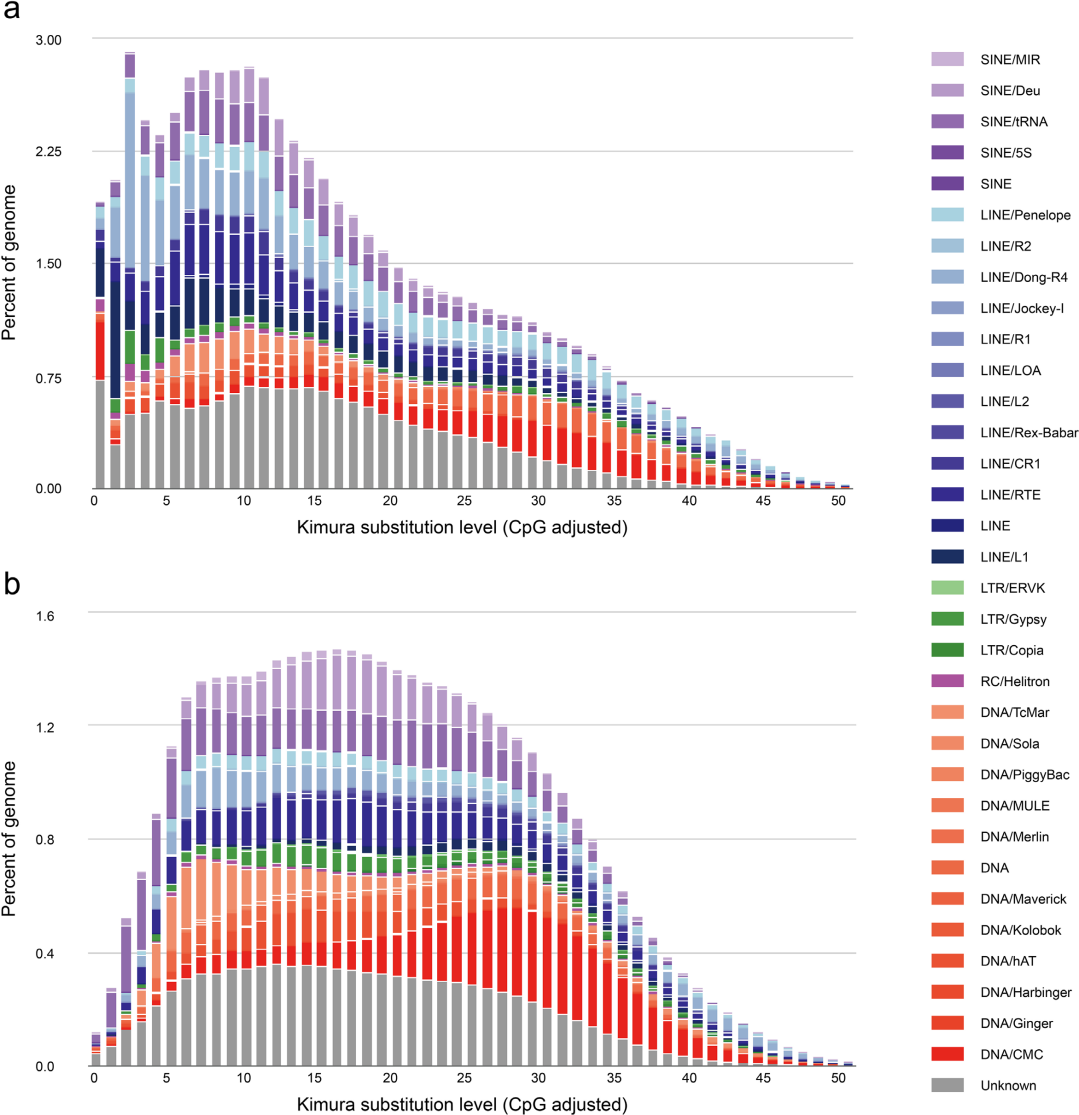
Transposable element (TE) accumulation history in the *Octopus* genomes. Kimura distance-based copy divergence analysis of TEs for **(a)***O. minor* and **(b)***O. bimaculoides. x*-axis, K-value; *y*-axis, genome coverage for each type of TE.

TEs are components of animal genomes, with major roles in genome rearrangements and evolution. Based on the mechanism of transposition, TEs are grouped into two main classes, class I retrotransposons, which are subdivided into long terminal repeats (LTRs) and non-LTR retrotransposons (e.g., LINEs and short interspersed elements [SINEs]), and class II DNA transposons [36]. We detected more TEs in the larger genome of *O. minor* than in the smaller genome of *O. bimaculoides*. Approximately half of the *O. minor* genome was composed of TEs (11,547,325 TEs; 44% of the genome), while one-third of the *O. bimaculoides* genome was composed of TEs (3,887,025 TEs; 35%) (Supplementary Table S11). The majority of class I retrotransposons in the *O. minor* genome were LINEs (10%), as was also the case in *O. bimaculoides* (9%), and the proportion of DNA transposons in *O. minor* (13%) was comparable to that in *O. bimaculoides* (12%). Interestingly, the *O. minor* genome had fewer SINEs (1,540 copies; 0.01%) and more rolling-circle (RC) helitrons (121,101 copies; 3.7%) than the *O. bimaculoides* genome (SINEs: 115,169 copies, 1.8%; RC helitron: 43,735 copies, 0.7%). A Kimura distance analysis revealed that the most frequent TE sequence divergence relative to the TE consensus sequence was ∼7%–10%, with an additional peak at 3% (Fig. 3a) compared to 16%–17% in the *O. bimaculoides* genome (Fig. 3b and Supplementary Table S11).

A more recent expansion of LINEs, without an increase in SINEs, was detected in the *O. minor* genome, while ancient copies of all four types of TEs and an ancient transposition burst of DNA transposons were observed in *O. bimaculoides*. Using the recent TE expansion in the *O. minor* genome, we correlated Jukes–Cantor distance measures with *d*_S_ and identified two unique expansion waves at 0.04 and 0.09 compared to the distribution of *O. bimaculoides* TEs (Supplementary Figs. S5 and S6). This suggests that a major expansion of TEs in the *O. minor* genome occurred 11 to 25 Mya, which is after the divergence of *O. minor* and *O. bimaculoides*.

## Conclusions

*Octopus minor* has developed morphological and physiological adaptations to match their unique mudflat habitats. In summary, we generated a high-quality sequence assembly for *O. minor* to elucidate the molecular mechanisms underlying their adaptations. In a direct comparison between the genomes of *O. minor* and *O. bimaculoides*, we discovered that they evolved recently and independently from the octopus lineage during the successful transition from an aquatic habitat to mudflats. We also found evidence suggesting that speciation in the genus *Octopus* is closely related to the gene family expansion associated with environmental adaptation. Finally, in addition to providing insights into the genome size increase via gene family expansion, the *O. minor* genome sequence also provides an essential resource for studies of Cephalopoda evolution.

## Availability of supporting data

The octopus (*O. minor*) genome project was deposited at the National Center for Biotechnology Information under BioProject number PRJNA421033. The whole-genome sequence was deposited in the Sequence Read Archive (SRA) database under accession number SRX3462978, and isoform sequence from PacBio sequencing data was deposited in the SRA database under accession numbers SRX3478495 and SRX3478496. Other supporting data, including annotations, alignments, and BUSCO results, are available in the *GigaScience* repository, GigaDB [37].

## Additional files

Fig. S1. Estimation of genome size of *O. minor* based on distribution of 17 k-mer frequency in raw sequencing reads.

Fig. S2. Genome size determination by flow cytometry. The flow cytometry analysis provides as estimation of Propidium iodide (PI) staining. Accepting a haploid genome size estimate of 2.81 Gb for Mouse (Assembly; GRCm38.p6), we estimate the genome size of O. minor to be 5.38 Gb.

Fig. S3. Blast top hit distribution.

Fig. S4. Composition of transposable elements in the regions of gene and intergenic sequence.

Fig. S5. Transposable elements Juke-cantor distance distribution.

Fig. S6. Transposable elements Juke-cantor distance distribution of *O. minor*.

Table S1. Statistics for SMRT sequencing for the *O. minor* genome sequencing.

Table S2. Isoform sequencing summary of transcriptome analysis of *O. minor* using PacBio RSII.

Table S3. Brief summary of gene statistics.

Table S4. Functional annotation statistics of transcriptome assembly.

Table S5. Summary of orthologous gene clusters analyzed in 14 species.

Table S6. CAFE gene family analysis results.

Table S7. Example of top 30 CAFE significantly expanded gene families.

Table S8. Example of top 30 CAFE significantly shrinked gene families.

Table S9. Top 30 expanded Pfam domains.

Table S10. Top 30 expanded EggNOG domains.

Table S11. Statistics of repeat analysis of the *O. minor* genome.

Table S12. Classifications and frequencies of transposable elements and other repeats.

Table S13. Classifications and frequencies of simple repeats.

Supplementary text commands

## Abbreviations

BLAST: Basic Local Alignment Search Tool; BUSCO: Benchmarking Universal Single-Copy Orthologs; LINEs: Long Interspersed Nuclear Elements; LTR: Long Terminal Repeat; Mya: Million years ago; PacBio: Pacific Biosciences; RC: Rolling Circle; SINEs: Short Interspersed Nuclear Elements; SMRT: Single-Molecular Real-Time; TEs: Transposable Elements.

## Ethics Statement

No specific permits were required for the described field studies, no specific permissions were required for these locations/activities, and the field studies did not involve endangered or protected species.

## Competing interests

The authors declare that they have no competing interests.

## Funding

This work was supported by a grant from National Marine Biodiversity Institute of Korea (MABIK)(2018M00900).

## Author contributions

H.S.A., H.P., and J.L. conceived the study. H.P., B.K., S.K., D.A., S.J., J.L., H.R., and S.L. performed genome sequencing, assembly, and annotation. S.J., Y.H., K.R., and S.C. performed experiments. J.S.Y., H.S.A., H.P., S.J., and J.L. advised and coordinated the study. B.K., S.K., D.A., and H.P. mainly wrote the paper. All authors contributed to writing and editing the manuscript and supplementary information and to producing the figures.

## Supplementary Material

Reviewer_1_Report_(Original_Submission) -- Joseph F. Ryan 6/18/2018 Reviewed

Reviewer_2_Report_(Original_Submission) -- Yi-Jyun Luo 6/24/2018 Reviewed

Reviewer_1_Report_Revision_1 -- Joseph F. Ryan 8/23/2018 Reviewed

Reviewer_2_Report_Revision_1 -- Yi-Jyun Luo 8/24/2018 Reviewed

Response_To_Reviewer_Comments_Revision_1.pdf

Response_To_Reviewer_Comments_(Original_Submission).pdf

GIGA-D-18-00174_Original_Submission.pdf

GIGA-D-18-00174_Revision_1.pdf

GIGA-D-18-00174_Revision_2.pdf

Supplemental Files
