# Supplementary material for: The genome of common long-arm octopus Octopus minor
Source: Gigascience. 2018 Sep 25;7(11):giy119. doi: 10.1093/gigascience/giy119 (PMC6279123; doi:10.1093/gigascience/giy119)
Supplement: GIGA-D-18-00174_Original_Submission.pdf [file giy119_giga-d-18-00174_original_submission.pdf]

GigaScience

The genome of common long-arm octopus Octopus minor

--Manuscript Draft--

**Manuscript Number:**	GIGA-D-18-00174
**Full Title:**	The genome of common long-arm octopus Octopus minor
**Article Type:**	Data Note
**Funding Information:**	MABIK (2018M00900) — Dr. Hye Suck An
**Abstract:**	Background: The common long-arm octopus (Octopus minor) is found in mudflats of subtidal zones and faces numerous environmental challenges. The ability to adapt its morphology and behavioural repertoire to diverse environmental conditions makes the species a promising model to understand genomic adaptation and evolution in cephalopods. Findings: The final genome assembly of O. minor is 5.09 Gb, with a contig N50 size of 197 kb and longest size of 3.027 Mb, from a total of 419 Gb raw reads generated using PacBio RS II platform. We identified 30,010 genes and 44.43% of the genome is composed of repeat elements. The genome-widw phylogenetic tree indicated the divergence time between O. minor and O. bimaculoides was estimated to be 43 million years ago (Mya) based on single-copy orthologous genes. In total, 178 gene families are expanded in O. minor in the 14 bilaterian animal species. Conclusion: We found that the O. minor genome was larger than that of closely related O. bimaculoides, and this difference could be explained by enlarged introns and recently diversified transposable elements. The high-quality O. minor genome assembly provides a valuable resource for understanding octopus genome evolution and the molecular basis of adaptations to mudflats.
**Corresponding Author:**	Hyun Park<br><br>KOREA, REPUBLIC OF
**Corresponding Author Secondary Information:**	
**Corresponding Author's Institution:**	
**Corresponding Author's Secondary Institution:**	
**First Author:**	Bo-Mi Kim
**First Author Secondary Information:**	
**Order of Authors:**	Bo-Mi Kim
	Seunghyun Kang
	Do-Hwan Ahn
	Seung-Hyun Jung
	Hwanseok Rhee
	Jong Su Yoo
	Jong-Eun Lee
	SeungJae Lee
	Yong-Hee Han
	Kyoung-Bin Ryu
	Sung-Jin Cho
	Hyun Park
	Hye Suck An

Powered by Editorial Manager® and ProduXion Manager® from Aries Systems Corporation

Order of Authors Secondary Information:	
**Additional Information:**	
**Question**	**Response**
Are you submitting this manuscript to a special series or article collection?	No
**Experimental design and statistics**<br><br>Full details of the experimental design and statistical methods used should be given in the Methods section, as detailed in our Minimum Standards Reporting Checklist. Information essential to interpreting the data presented should be made available in the figure legends.<br><br>Have you included all the information requested in your manuscript?	Yes
**Resources**<br><br>A description of all resources used, including antibodies, cell lines, animals and software tools, with enough information to allow them to be uniquely identified, should be included in the Methods section. Authors are strongly encouraged to cite Research Resource Identifiers (RRIDs) for antibodies, model organisms and tools, where possible.<br><br>Have you included the information requested as detailed in our Minimum Standards Reporting Checklist?	Yes
**Availability of data and materials**<br><br>All datasets and code on which the conclusions of the paper rely must be either included in your submission or deposited in publicly available repositories (where available and ethically appropriate), referencing such data using a unique identifier in the references and in the "Availability of Data and Materials" section of your manuscript.<br><br>Have you have met the above requirement as detailed in our Minimum Standards Reporting Checklist?	Yes

Powered by Editorial Manager® and ProduXion Manager® from Aries Systems Corporation

manuscript Click here to download Manuscript Kim etal.docx

The genome of common long-arm octopus *Octopus minor*

Bo-Mi Kim [a,†], Seunghyun Kang [a,†], Do-Hwan Ahn [a,†], Seung-Hyun Jung [b,†], Hwanseok Rhee [c,†], Jong Su Yoo [b], Jong-Eun Lee [c], SeungJae Lee [c], Yong-Hee Han [d], Kyoung-Bin Ryu [d], Sung-Jin Cho [d,*], Hyun Park [a,e,*], Hye Suck An [b,*]

Affiliations

[a] Unit of Polar Genomics, Korea Polar Research Institute(KOPRI), Incheon 21990, Korea
[b] Department of Genetic Resources Research, National Marine Biodiversity Institute of Korea (MABIK), Janghang-eup, Seochun-gun, Chungchungnam-do 33662, Korea
[c] Genomics Lab, Cluster Center, DNA Link, Inc., 150, Bugahyeon-ro, Seodaemun-gu, Seoul 03759, Korea
[d] School of Biological Sciences, College of Natural Sciences, Chungbuk National University, Cheongju, Chungbuk 28644, Korea
[e] Polar Sciences, University of Science & Technology, Yuseong-gu, Daejeon 34113, Korea

---

[*]Co-corresponding author:

School of Biological Sciences, College of Natural Sciences, Chungbuk National University, Cheongju, Chungbuk 28644, Korea; E-mail address: sjchobio@chungbuk.ac.kr (S. Cho)

Unit of Polar Genomics, Korea Polar Research Institute, Incheon 21990, Korea; E-mail address: hpark@kopri.re.kr (H. Park)

Department of Genetic Resources Research, National Marine Biodiversity Institute of Korea (MABIK), Janghang-eup, Seochun-gun, Chungchungnam-do 33662, Korea; E-mail address: mgran@mabik.re.kr H.S. An)

[†]These authors contributed equally to this work.

Abstract

Background: The common long-arm octopus (*Octopus minor*) is found in mudflats of subtidal zones and faces numerous environmental challenges. The ability to adapt its morphology and behavioural repertoire to diverse environmental conditions makes the species a promising model to understand genomic adaptation and evolution in cephalopods. **Findings**: The final genome assembly of *O. minor* is 5.09 Gb, with a contig N50 size of 197 kb and longest size of 3.027 Mb, from a total of 419 Gb raw reads generated using PacBio RS II platform. We identified 30,010 genes and 44.43% of the genome is composed of repeat elements. The genome-widw phylogenetic tree indicated the divergence time between *O. minor* and *O. bimaculoides* was estimated to be 43 million years ago (Mya) based on single-copy orthologous genes. In total, 178 gene families are expanded in *O. minor* in the 14 bilaterian animal species. **Conclusion**: We found that the *O. minor* genome was larger than that of closely related *O. bimaculoides,* and this difference could be explained by enlarged introns and recently diversified transposable elements. The high-quality *O. minor* genome assembly provides a valuable resource for understanding octopus genome evolution and the molecular basis of adaptations to mudflats.

Key words:

Octopus genome, Cephalopods, adaptation and evolution, long-read sequencing

Introduction

Cephalopods (*e.g.* cuttlefish, nautilus, octopus, and squid) belong to the phylum Mollusca. As advanced invertebrates, cephalopods have interesting biological characteristics, such as an extraordinary life-history plasticity, rapid growth, short lifespan, large brain, and sophisticated sense organs with a complex nervous system[1]. The ability to adapt their morphology and behavioural repertoire to diverse environmental conditions and capacity for learning and memory are common traits in cephalopods, but have rarely been observed in other invertebrates[2]. Many cephalopod species have been considered for fisheries and are promising candidates for aquaculture. There are an estimated 1,000 cephalopod species (~700 known marine-living species), and octopods are among the most well-known representatives of the class, including over 150 species worldwide[3]. Studies have evaluated the biological machinery underlying the fundamental nervous system functions, strong behavioural plasticity, and learning ability in octopods[4, 5].

Octopus minor (Sasaki, 1920), also known as the common long-arm octopus, is a benthic littoral species, and is a major commercial fishery product with a high annual yield[6]. *O. minor* is relatively small and possesses a shorter life cycle (approximately 1 year), thinner arms, and a lower ratio between head size and arm length compared to those of other octopus species (**Fig. 1a**). The species is widely distributed in Northeast Asia, particularly in coastal regions of South Korea, China, and Japan (**Fig. 1b**). Most *O. minor* habitats are mud and mud sand in well-developed mudflats of coastal regions; they spawn in holes on the mudflat by digging with the whole body. As an important economic cephalopod in South Korea, fishermen normally catch *O. minor* by digging a hole in the mudflat with shovels. Thus, they are subjected to the harsh environmental conditions of mudflats, including diurnal temperature changes, steep salinity and pH gradients, desiccation, wave action and tides, oxygen availability, and interrupted feeding. Owing to the ability of *O. minor* to tolerate environmental fluctuations, it is a promising organism for studies of the molecular basis of plasticity and mechanisms underlying adaptation to harsh environmental conditions, although relevant information is scarce. To make full use of this emerging cephalopod model system and to understand the interesting features of *O. minor*, including its plasticity in mudflats and genetic evolution, a high-quality reference genome is required.

The published genome and multiple transcriptomes of the California two-spot octopus *Octopus bimaculoides* have provided valuable information on genomic traits (*e.g.* gene family expansion, genome rearrangements, and transposable element activity) related to the evolution

of neural complexity and morphological innovations[7]. In this study, we report a high-quality genome assembly and annotation for *O. minor*. We compare the genomes of *O. minor* and *O. bimaculoides* and provide evidence that the expansion of genes and/or gene families is related to adaptation to the harsh environmental conditions of mudflats.

Genome sequencing and annotation

O. minor genomic DNA was extracted from leg muscle tissues. The average coverage of SMRT sequences was ~76-fold using P6-C4 sequence chemistry from genomic DNA libraries. The average subread length was 9.2 kb (Supplementary Table S1). For genome size estimation, a k-mer analysis was performed using Jellyfish[8] with paired-end sequences of the genomic DNA libraries. The *O. minor* genome was estimated to be 5.1 Gb (Supplementary Figs. S1 and S2). The *de novo* assembly generated using FALCON-Unzip assembler was 5.09 Gb with 41,584 contigs[9]. Finally, evaluation of the genome completeness was checked using BUSCO[10] (Supplementary Table S2).

Total RNA was extracted from thirteen tissues (brain, branchial heart, buccal mass, eye, heart, kidney, liver, ovary, poison gland, siphon, skin, and suckers) using the RNeasy Mini Kit (Qiagen, Hilden, Germany) according to the manufacturer's instructions. RNA quality was confirmed using an Agilent Bioanalyzer™. Isoform sequencing was performed using pooled RNA from thirteen organs. Library construction and sequencing were performed using Pacbio RS II (Supplementary Table S3). The SMRTbell library for Iso-seq was sequenced using 16 SMRT cells (1–2 kb, three cells; 2–3 kb, six cells; and 3–6 kb, seven cells). Reads were identified using the SMRT Analysis ver. 2.3 RS_IsoSeq.1 classification protocol. All full-length reads derived from the same isoform were clustered and consensus sequences were polished using the TOFU pipeline (isoseq-tofu)[11]. Additionally, chimeras of consensus sequences were removed.

MAKER was used for genome annotation[12]. First, repetitive elements were identified using RepeatMasker[13]. A *de novo* repeat library was constructed using RepeatModeler (ver. 1.0.3)[14], including RECON[15] and RepeatScout[16], with default parameters. Consensus sequences and classification information for each repeat family were generated, and tandem repeats, including simple repeats, satellites, and low-complexity repeats, were predicted using Tandem Repeats Finder[11]. This masked genome sequence was used for *ab initio* gene prediction with SNAP software[17]; subsequently, alignments of expressed sequence tags with BLASTn and protein information from tBLASTx were included. The *de novo* repeat library of

O. minor from RepeatModeler was used for RepeatMasker; proteins from sequenced molluscs (*L. gigantea*, *C. gigas*, and *Aplysia californica*) and an octopus species (*O. bimaculoides*) were included in the analysis. Transcriptome assembly results were used for expressed sequence tags. Next, MAKER polished the alignments using Exonerate, which provided integrated information for SNAP annotation. Using MAKER, the final gene model was selected and revised considering all information. A total of 30,010 *O. minor* genes were predicted using MAKER. The Infernal software package (ver. 1.1)[18] and covariance models from the Rfam[19] database were used to identify other non-coding RNAs in the *O. minor* scaffold. Putative tRNA genes were identified using tRNAscan-SE[20]. tRNAscan-SE uses a covariance model that scores candidates based on their sequence and predicted secondary structures.

The mean size of *O. minor* genes was 23.6 kb, with an average intron length of 5.4 kb (4.2 introns per gene) (Supplementary Table S4). The *O. minor* genome contained 30,010 protein-coding genes (Table 1), of which 96% were annotated based on known proteins in public databases, and 79% were similar to *O. bimaculoides* genes (Supplementary Table S5).

Comparative genomic analyses and duplicate genes

To resolve gene family evolution in the *O. minor* genome, we classified orthologous gene clusters (Supplementary Table S6) from 14 species and found evidence for the recent expansion of low-copy gene duplicates and the expansion of large gene families. Orthologous groups were identified using both OrthoMCL[21] and Pfam[22] domain assignments. OrthoMCL generated a graphical representation of sequence relationships, which was then divided into subgraphs using the Markov Clustering Algorithm (MCL) from multiple eukaryotic genomes[21]. The standard parameters and options of OrthoMCL were used for all steps, together with the genomes of 14 species (Supplementary Table S6). For *O. minor*, the coding sequence from the MAKER annotation pipeline was used. To construct a phylogenetic tree and estimate the divergence time, 202 1:1:1 single-copy orthologous genes were used. Using the Probabilistic Alignment Kit (PRANK)[23], protein-coding genes were aligned with the codon alignment option, and poorly aligned regions with gaps were eliminated using Gblock[24] with a codon model. A maximum-likelihood tree was built using RAxML[25] with 1,000 bootstrap replicates, and the divergence time was calibrated using TimeTree[26]. The average gene gain-loss was identified using CAFÉ 4.0[27].

Sequence divergence was estimated by calculating d_S values using the yn00 program from the PAML package[28]. The Jukes–Cantor distances were adjusted using the Jukes–Cantor

formula d_{XY} = -(3/4)ln(1-4/3D), where D is the proportion of nucleotide differences between the sequences. The time estimation was calibrated by assuming d_S of ~1 is 135 million years[7].

Gene family analyses of specific genes of interest were manually curated using manual gene search methods. Gene or gene family targets identified in the genomes of *Octopus bimaculoides*, *Crassostrea gigas*, *Lottia gigantea*, *Capitella teleta*, and *Homo sapiens* were directly mapped to the *O. minor* genome database by a local BLAST analysis. Alignments were generated using Clustal Omega (ClustalO)[29] and Multiple Sequence Comparison by Log-Expectation (MUSCLE)[30], and phylogenetic trees were built using FastTree[31] or RAxML with 1,000 bootstrap replicates.

A statistical analysis of the changes in gene family sizes indicated significantly greater gene family expansion in *O. minor* (178 gene families) compared to other species, *e.g.* interleukin-17, G protein-coupled receptor (GPCR) proteins, Zinc-finger of C2H2 type, heat shock protein (HSP) 70 proteins, and cadherin-like domains (Supplementary Tables S7–S9). The divergence time between *O. minor* and *O. bimaculoides* was estimated to be 43 million years ago (Mya) based on single-copy orthologous genes (Fig. 2a) Further, Pfam domain and EggNOG metazoan database searches consistently showed the expansion of gene families, including the cadherin and protocadherin domains and interleukin-17 (Fig. 2b and Supplementary Tables S10 and S11).

Transposable element annotation and expansions

The *O. minor* genome was larger than that of *O. bimaculoides* (2.7 Gb), with a high level of repetitive sequences (44.43%) (Supplementary Tables S12–S14). Repeats accounted for 44% (2.262 Gb) of the assembly, and were dominated by simple repeats (14.7%) and TEs, especially DNA transposons and long interspersed elements (LINEs), which were more abundant in the *O. minor* genome than in the *O. bimaculoides* genome. In an analysis of genes (i.e. exons and introns) and intergenic sequences, TEs were highly distributed in the intergenic sequence regions in both species (Supplementary Fig. S4). In particular, TE accumulation in intergenic sequence regions was significantly greater in *O. minor* than in O. *bimaculoides*. The larger gene size and higher repeat content may explain the larger genome of *O. minor* compared with *O. bimaculoides*.

TEs are crucial components of animal genomes, with major roles in genome rearrangements and evolution. Based on the mechanism of transposition, TEs are grouped into two main classes, class I retrotransposons, which are subdivided into long terminal repeats

(LTRs) and non-LTR retrotransposons [*e.g.* LINEs and short interspersed elements (SINEs)], and class II DNA transposons[32]. We detected more TEs in the larger genome of *O. minor* than in the smaller genome of *O. bimaculoides*. Approximately half of the *O. minor* genome was composed of TEs (11,547,325 TEs; 44% of the genome), while one-third of the *O. bimaculoides* genome was composed of TEs (3,887,025 TEs; 35%) (Supplementary Table S12). The majority of class I retrotransposons in the *O. minor* genome were LINEs (10%), as was also the case in *O. bimaculoides* (9%), and the proportion of DNA transposons in *O. minor* (13%) was comparable to that in *O. bimaculoides* (12%). Interestingly, the *O. minor* genome had fewer SINEs (1,540 copies; 0.01%) and more rolling-circle (RC)-Helitrons (121,101 copies; 3.7%) than the *O. bimaculoides* genome (SINEs: 115,169 copies, 1.8%; RC-Helitron: 43,735 copies, 0.7%). A Kimura distance analysis revealed that the most frequent TE sequence divergence relative to the TE consensus sequence was ~7–10%, with an additional peak at 3% (Fig. 3a), compared to 16–17% in the *O. bimaculoides* genome (Fig. 3b and Supplementary Table S12).

A more recent expansion of LINEs, without an increase in SINEs, was detected in the *O. minor* genome, while ancient copies of all four types of TEs and an ancient transposition burst of DNA transposons were observed in *O. bimaculoides*. Using the recent TE expansion in the *O. minor* genome, we correlated Jukes–Cantor distance measures with d_S and identified two unique expansion waves at 0.04 and 0.09 compared to the distribution of *O. bimaculoides* TEs (Supplementary Figs. S5 and S6). This suggests that a major expansion of TEs in the *O. minor* genome occurred 11 to 25 Mya, which is after the divergence of *O. minor* and *O. bimaculoides*.

Conclusions

O. minor has developed morphological and physiological adaptations to match their unique mudflat habitats. In summary, we generated a high-quality sequence assembly for *O. minor* to elucidate the molecular mechanisms underlying their adaptations. In a direct comparison between the genomes of *O. minor* and *O. bimaculoides*, we discovered that they evolved recently and independently from the octopus lineage during the successful transition from an aquatic habitat to mudflats. We also found evidence suggesting that speciation in the genus *Octopus* is closely related to the gene family expansion associated with environmental adaptation. Finally, in addition to providing insights into the genome size increase via gene family expansion, the *O. minor* genome sequence also provides an essential resource for studies of Cephalopoda evolution.

Availability of supporting data

The octopus (*O. minor*) genome project was deposited at NCBI under BioProject number PRJNA421033. The whole-genome sequence was deposited in the Sequence Read Archive (SRA) database under accession number SRX3462978, and isoform sequence from PacBio sequencing data were deposited in the SRA database under accession numbers SRX3478495 and SRX3478496. Other supporting data, including annotations, alignments, and BUSCO results, are available in the GigaScience repository, GigaDB [---].

Additional files

Fig. S1. Estimation of genome size of *O. minor* based on distribution of 17 k-mer frequency in raw sequencing reads.

Fig. S2. Genome size determination by flow cytometry. The flow cytometry analysis provides as estimation of Propidium iodide (PI) staining. Accepting a haploid genome size estimate of 2.81 Gb for Mouse (Assembly; GRCm38.p6), we estimate the genome size of O. minor to be 5.38 Gb.

Fig. S3. Blast top hit distribution.

Fig. S4. Composition of transposable elements in the regions of gene and intergenic sequence.

Fig. S5. Transposable elements Juke-cantor distance distribution.

Fig. S6. Transposable elements Juke-cantor distance distribution of *O. minor*.

Table S1. Statistics for SMRT sequencing for the *O. minor* genome sequencing.

Table S2. Benchmarking Universal Single-Copy Orthologs (BUSCO) evaluated for the completeness of the *O. minor* genome assembly.

Table S3. Isoform sequencing summary of transcriptome analysis of *O. minor* using PacBio RSII.

Table S4. Brief summary of gene statistics.

Table S5. Functional annotation statistics of transcriptome assembly.

Table S6. Summary of orthologous gene clusters analyzed in 14 species.

Table S7. CAFÉ gene family analysis results.

Table S8. Example of top 30 CAFÉ significantly expanded gene families.

Table S9. Example of top 30 CAFÉ significantly shrinked gene families.

Table S10. Top 30 expanded Pfam domains.

Table S11. Top 30 expanded EggNOG domains.

Table S12. Statistics of repeat analysis of the *O. minor* genome.

Table S13. Classifications and frequencies of transposable elements and other repeats.

Table S14. Classifications and frequencies of simple repeats.

Acknowledgements

We thank Jong Won Han and Ha Yeun Song of the National Marine Biodiversity Institute of Korea (MABIK) for the sampling of 18 tissues used for transcriptome assembly, as well as Keekwang Kim of Chungnam National University and Kun-Hee Kim of Chonnam National University for their devotion to estimate the genome size of *O. minor* by flow cytometry. We also thank Jeollanam-Do Oceans & Fisheries Science Institute for providing octopus embryos.

Funding

This work was supported by grants (2018M00900) from MABIK.

Competing interests

The authors declare that they have no competing interests.

Author contributions

H.S.A., H.P., and J.L. conceived the study. H.P., B.K., S.K., D.A., S.J., J.L., H.R., and S.L. performed genome sequencing, assembly, and annotation. S.J., Y.H., K.R., and S.C. performed experiments. J.S.Y., H.S.A., H.P., S.J., and J.L. advised and coordinated the study. B.K., S.K., D.A., and H.P. mainly wrote the paper. All authors contributed to writing and editing the manuscript and supplementary information and producing the figures.

References

1. Boyle P and Rodhouse P. Cephalopods: ecology and fisheries. Oxford: Blackwell Science Ltd; 2005.
2. Hanlon RT and Messenger JB. Cephalopod behaviour. Cambridge: Cambridge University Press; 1998.
3. Guzik MT, Norman MD and Crozier RH. Molecular phylogeny of the benthic shallow-water octopuses (Cephalopoda: Octopodinae). Mol Phylogen Evol. 2005;

37 1:235-48.

4. Hochner B, Shomrat T and Fiorito G. The octopus: a model for a comparative analysis of the evolution of learning and memory mechanisms. Biol Bull. 2006;210 3:308-17.

5. Mather JA. Cephalopod consciousness: behavioural evidence. Conscious Cogn. 2008;17 1:37-48.

6. MIFAFF. Food, Agriculture, Forestry and Fisheries statistical yearbook. Seoul: Forestry and Fisheries (MIFAFF) Press; 2012.

7. Albertin CB, Simakov O, Mitros T, Wang ZY, Pungor JR, Edsinger-Gonzales E, et al. The octopus genome and the evolution of cephalopod neural and morphological novelties. Nature. 2015;524 7564:220-4.

8. Marçais G and Kingsford C. A fast, lock-free approach for efficient parallel counting of occurrences of k-mers. Bioinformatics. 2011;27 6:764-70.

9. Chin C-S, Peluso P, Sedlazeck FJ, Nattestad M, Concepcion GT, Clum A, et al. Phased diploid genome assembly with single molecule real-time sequencing. Nat Methods. 2016;13 12:1050.

10. Simão FA, Waterhouse RM, Ioannidis P, Kriventseva EV and Zdobnov EM. BUSCO: assessing genome assembly and annotation completeness with single-copy orthologs. Bioinformatics. 2015;31 19:3210-2.

11. Gordon SP, Tseng E, Salamov A, Zhang J, Meng X, Zhao Z, et al. Widespread polycistronic transcripts in fungi revealed by single-molecule mRNA sequencing. PLoS ONE. 2015;10 7:e0132628.

12. Holt C and Yandell M. MAKER2: an annotation pipeline and genome-database management tool for second-generation genome projects. BMC bioinformatics. 2011;12 1:491.

13. Smit AFA HR, Green, P. RepeatMasker Open-3.0. 1996-2004 (http://www.RepeatMakser.org).

14. Bao Z and Eddy SR. Automated de novo identification of repeat sequence families in sequenced genomes. Genome research. 2002;12 8:1269-76.

15. Price AL, Jones NC and Pevzner PA. De novo identification of repeat families in large genomes. Bioinformatics. 2005;21 suppl_1:i351-i8.

16. Benson G. Tandem repeats finder: a program to analyze DNA sequences. Nucleic acids research. 1999;27 2:573.

17. Korf I. Gene finding in novel genomes. BMC bioinformatics. 2004;5 1:59.
18. Nawrocki EP, Kolbe DL and Eddy SR. Infernal 1.0: inference of RNA alignments. Bioinformatics. 2009;25 10:1335-7.
19. Gardner PP, Daub J, Tate J, Moore BL, Osuch IH, Griffiths-Jones S, et al. Rfam: Wikipedia, clans and the “decimal” release. Nucleic Acids Res. 2010;39 suppl_1:D141-D5.
20. Lowe TM and Eddy SR. tRNAscan-SE: a program for improved detection of transfer RNA genes in genomic sequence. Nucleic acids research. 1997;25 5:955.
21. Li L, Stoeckert CJ, Jr. and Roos DS. OrthoMCL: identification of ortholog groups for eukaryotic genomes. Genome Res. 2003;13 9:2178-89.
22. Finn RD, Bateman A, Clements J, Coggill P, Eberhardt RY, Eddy SR, et al. Pfam: the protein families database. Nucleic Acids Res. 2013;42 D1:D222-D30.
23. Löytynoja A and Goldman N. An algorithm for progressive multiple alignment of sequences with insertions. Proceedings of the National Academy of Sciences of the United States of America. 2005;102 30:10557-62.
24. Castresana J. Selection of conserved blocks from multiple alignments for their use in phylogenetic analysis. Molecular biology and evolution. 2000;17 4:540-52.
25. Stamatakis A. RAxML version 8: a tool for phylogenetic analysis and post-analysis of large phylogenies. Bioinformatics. 2014;30 9:1312-3.
26. Hedges SB, Dudley J and Kumar S. TimeTree: a public knowledge-base of divergence times among organisms. Bioinformatics. 2006;22 23:2971-2.
27. Han MV, Thomas GW, Lugo-Martinez J and Hahn MW. Estimating gene gain and loss rates in the presence of error in genome assembly and annotation using CAFE 3. Molecular biology and evolution. 2013;30 8:1987-97.
28. Yang Z. PAML 4: phylogenetic analysis by maximum likelihood. Molecular biology and evolution. 2007;24 8:1586-91.
29. Sievers F, Wilm A, Dineen D, Gibson TJ, Karplus K, Li W, et al. Fast, scalable generation of high-quality protein multiple sequence alignments using Clustal Omega. Molecular systems biology. 2011;7 1:539.
30. Edgar RC. MUSCLE: multiple sequence alignment with high accuracy and high throughput. Nucleic Acids Res. 2004;32 doi:10.1093/nar/gkh340.

31. Price MN, Dehal PS and Arkin AP. FastTree 2–approximately maximum-likelihood trees for large alignments. PloS one. 2010;5 3:e9490.

32. Wicker T, Sabot F, Hua-Van A, Bennetzen JL, Capy P, Chalhoub B, et al. A unified classification system for eukaryotic transposable elements. Nature Reviews Genetics. 2007;8 12:973-82.

Figure legends

Figure 1: Common long-arm octopus (*Octopus minor*). **a** Habitat structure of mudflats and phenotypic differences between *Octopus minor* and *O. bimaculoides*. *O. minor* has a smaller body size and possesses longer, thinner arms than those of *O. bimaculoides*. **b** The distribution of *O. minor* is shown in dark red. The distribution map was updated from Roper *et al*. (1984).

Figure 2: Gene family analysis for 14 bilaterian animal species. **a** Divergence times and gene family gain-and-loss analysis of 14 bilaterian animal species. **b** Heat map of expanded Pfam domains in the *O. minor* genome. OM, *Octopus minor*; OB, *Octopus bimaculoides*; LG, *Lottia gigantea*; CG, *Crassostrea gigas*; PF, *Pinctada fucata*; LA, *Lingula anatina*; CT, *Capitella teleta*; HR, *Helobdella robusta*; CE, *Caenorhabditis elegans*; DM, *Drosophila melanogaster*; DP, *Daphnia pulex*; SP, *Strongylocentrotus purpuratus*; MM, *Mus musculus*; HS, *Homo sapiens.*

Figure 3: Transposable element (TE) accumulation history in the *Octopus* genomes. Kimura distance-based copy divergence analysis of TEs for **a**, *O. minor* and **b**, *O. bimaculoides*. *x*-axis, K-value; *y*-axis, genome coverage for each type of TE.

 Click here to download Table Table1.docx

Table 1 Overview of the assembly and annotation of the *Octopus minor* genome.

Total length (bp)	5,090,349,614
Number of contigs	41,584
Contig N50 (bp)	196,941
Largest contigs (bp)	3,027,443
GC content (%)	36.33
Number of protein-coding genes	30,010

Click here to download Figure Fig 1.jpg

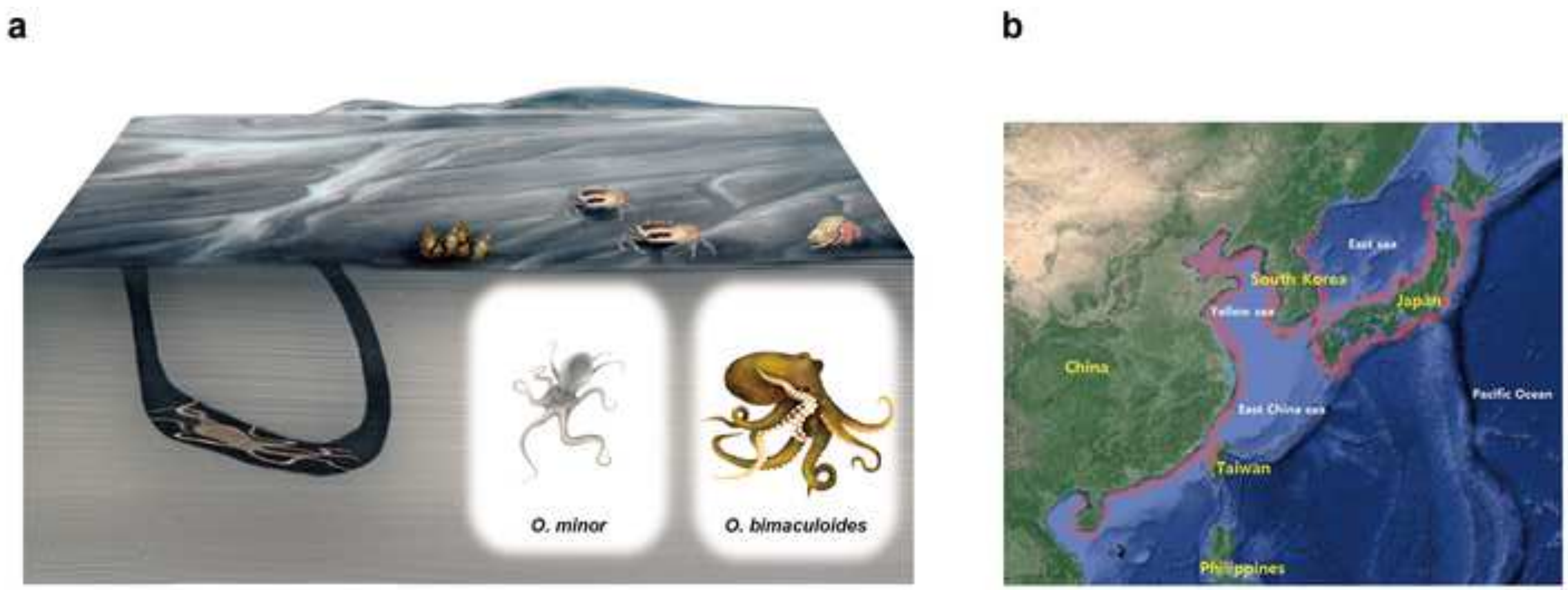

figure 2

Click here to download Figure Fig 2.jpg

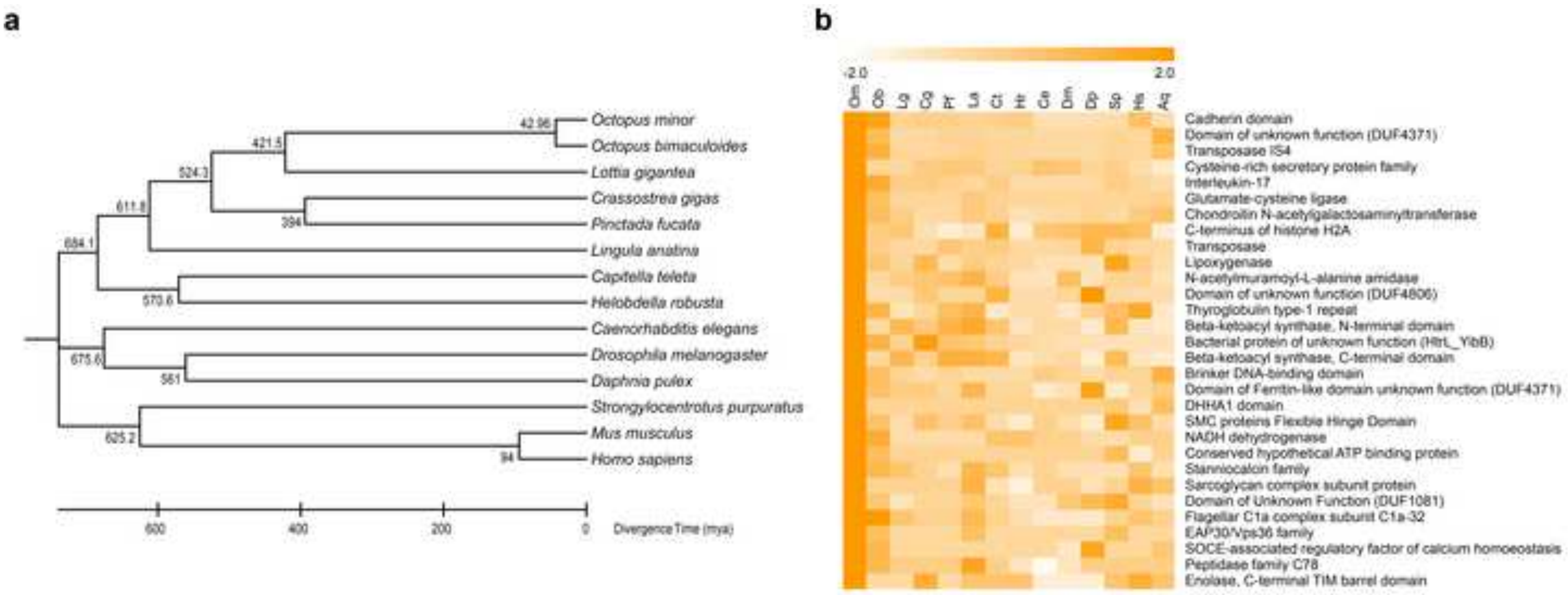

figure 3

Click here to download Figure Fig 3.jpg

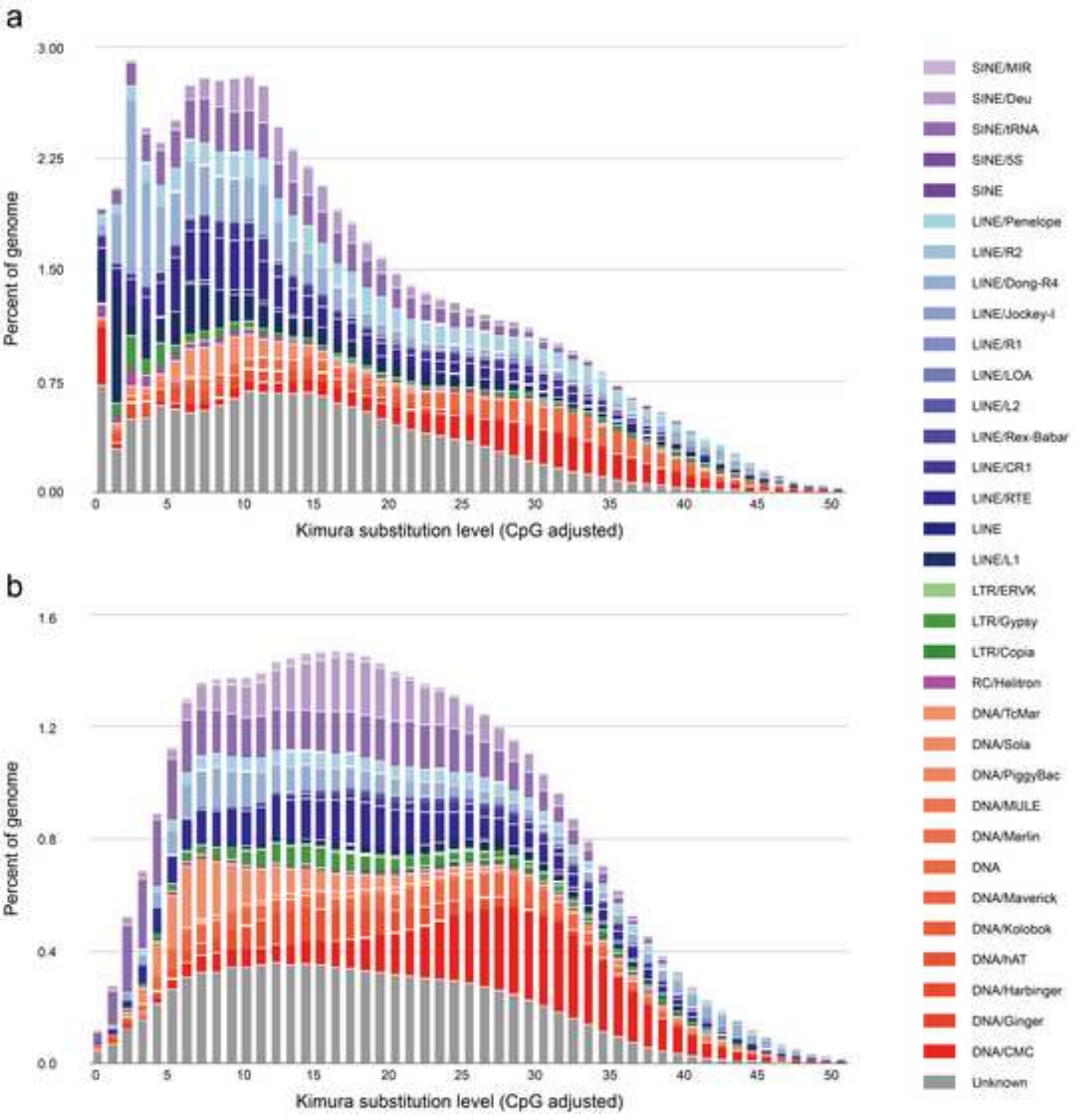

supplementary figs

Click here to access/download
Supplementary Material
GIGA_Additional file 1_figure.docx

supplementary tables

Click here to access/download
Supplementary Material
GIGA_Additional file 1_Table.docx
