# Supplementary material for: The genome of common long-arm octopus Octopus minor
Source: Gigascience. 2018 Sep 25;7(11):giy119. doi: 10.1093/gigascience/giy119 (PMC6279123; doi:10.1093/gigascience/giy119)
Supplement: Supplemental Files [file giy119_supplemental_files.zip › GIGA_Additional file 1_Table_R2.docx]

**Table S1. Statistics for SMRT sequencing for the *O. minor* genome sequencing.**

| **Number of SMRTcells** | 387 |
| --- | --- |
| **Polymerase Read** |  |
| Total number of bases (bp) | 418,704,242,425 |
| Number of reads | 30,807,673 |
| Mean subread length (bp) | 13,590 |
| N50 (bp) | 18,714 |
| Read quality | 0.84 |
| **Subread** |  |
| Total number of bases (bp) | 418,034,586,504 |
| Number of reads | 45,099,251 |
| Mean subread length (bp) | 9,269 |
| N50 (bp) | 14,013 |
| **Genome Coverage (5.1G)** | 82× |

**Table S2. Isoform sequencing summary of transcriptome analysis of *O. minor* using PacBioRS II.**

| Number of cells | 16 |
| --- | --- |
| Number of full-length non-chimeric reads | 549,569 |
| Number of consensus isoforms | 183,155 |
| Average consensus isoforms read length (bp) | 2,788 |
| Number of polished low-quality isoforms | 88,240 |
| Number of polished high-quality isoforms | 94,915 |
| Filtered high-quality isoforms | 94,021 |

*Isoform sequencing were used pooled RNA from 13 organs, brain, branchial heart, buccal mass, eye, heart, kidney, liver, ovary, poison gland, siphon, skin and suckers.

**Table S3. Brief summary of gene statistics.**

|  | ***O. minor*** |
| --- | --- |
| Protein-coding genes | 30,010 |
| Sum of gene length (bp) | 707,898,612 |
| Average length of genes (bp) | 23,589 |
| **Exon** |  |
| Number of exon | 154,580 |
| Average length of exon (bp) | 224 |
| Average number of exon per gene | 5.15 |
| Sum of total exon length (bp) | 34,650,052 |
| **Intron** |  |
| Number of intron | 124,570 |
| Average length of intron (bp) | 5,405 |
| Average number of intron per gene | 4.15 |
| Sum of total intron length (bp) | 673,248,560 |

**Table S4. Functional annotation statistics of transcriptome assembly.**

| Functional database | Count |
| --- | --- |
| BlastP (nr) | 21,870 |
| Interpro | 15,188 |
| GO | 12,035 |
| TreEMBL | 28,876 |
| SwissPort | 26,869 |
| **Total unigenes** | **30,010** |

**Table S5. Summary of orthologous gene clusters analyzed in 14 species.**

| Species  IDs | Species Name | Reference | No. of  coding genes | No. of gene families | No. of genes in gene families | No. of orphan genes | No. of unique gene families | Average No. of genes in gene families |
| --- | --- | --- | --- | --- | --- | --- | --- | --- |
| OM | *Octopus minor* | This study | 30,010 | 11,740 | 24,448 | 5562 | 963 | 2.08 |
| OB | *Octopus bimaculoides* | Ensembl Metazoa release 34 | 33,609 | 12,995 | 18,934 | 14675 | 456 | 1.46 |
| LG | *Lottia gigantean* | Ensembl Metazoa release 34 | 23,340 | 11,485 | 17,937 | 5403 | 791 | 1.56 |
| CG | *Crassostrea gigas* | Ensembl Metazoa release 34 | 26,089 | 11,874 | 19,227 | 6861 | 1,188 | 1.62 |
| PF | *Pinctada fucata* | http://marinegenomics.oist.jp/pearl/viewer/download?project_id=36 | 31,477 | 13,112 | 23,098 | 8379 | 1,609 | 1.76 |
| LA | *Lingula anatina* | Ensembl Metazoa release 34 | 34,105 | 13,385 | 28,425 | 5680 | 3,181 | 2.12 |
| CT | *Capitella teleta* | Ensembl Metazoa release 34 | 32,175 | 11,944 | 24,661 | 7514 | 1,862 | 2.06 |
| HR | *Helobdella robusta* | Ensembl Metazoa release 34 | 23,432 | 7,725 | 14,292 | 9140 | 598 | 1.85 |
| CE | *Caenorhabditis elegans* | Ensembl Metazoa release 34 | 20,362 | 5,874 | 12,800 | 7562 | 1,403 | 2.18 |
| DM | *Drosophila melanogaster* | Ensembl release 70 | 13,937 | 6,491 | 9,111 | 4826 | 544 | 1.40 |
| DP | *Daphnia pulex* | Ensembl Metazoa release 34 | 30,895 | 8,865 | 22,790 | 8105 | 2,171 | 2.57 |
| SP | *Strongylocentrotus purpuratus* | Ensembl Metazoa release 34 | 28,549 | 9,858 | 21,871 | 6678 | 1,471 | 2.22 |
| MM | *Mus musculus* | Ensembl release 70 | 22,808 | 14,894 | 21,446 | 1362 | 354 | 1.44 |
| HS | *Homo sapiens* | Ensembl release 70 | 20,477 | 14,736 | 19,321 | 1154 | 156 | 1.31 |

**Table S6. CAFE gene family analysis results.**

| Species | Expanded families | Genes gained | No change | Contracted families | Genes lost |
| --- | --- | --- | --- | --- | --- |
| *Octopus minor* | 2,303 (178) | 4,382 | 21,840 | 2,375 (55) | 2,559 |
| *Octopus bimaculoides* | 654 (92) | 1,386 | 25,057 | 807 (131) | 1,375 |
| *Lottia gigantea* | 1,136 (40) | 2,274 | 20,372 | 5,010 (1) | 5,050 |
| *Crassostrea gigas* | 1,429 (35) | 2,489 | 22,807 | 2,282 (0) | 2,353 |
| *Pinctada fucata* | 1,741 (67) | 3,600 | 23,324 | 1,453 (1) | 1,487 |
| *Lingula anatina* | 4,409 (64) | 7,233 | 13,607 | 8,502 (3) | 8,569 |
| *Strongylocentrotus purpuratus* | 2,149 (62) | 4,809 | 6,064 | 18,305 (0) | 18,340 |
| *Capitella teleta* | 1,583 (69) | 4,059 | 15,582 | 9,353 (0) | 9,385 |
| *Helobdella robusta* | 903 (29) | 2,084 | 13,282 | 12,333 (1) | 12,476 |
| *Caenorhabditis elegans* | 428 (7) | 995 | 22,424 | 3,666 (1) | 3,830 |
| *Drosophila melanogaster* | 522 (4) | 885 | 23,787 | 2,209 (0) | 2,300 |
| *Daphnia pulex* | 858 (58) | 2,976 | 24,256 | 1,404 (0) | 1,443 |
| *Mus musculus* | 670 (28) | 1,094 | 25,607 | 241 (10) | 298 |
| *Homo sapiens* | 535 (17) | 799 | 25,729 | 254 (15) | 347 |

**Table S7. Example of top 30 CAFE significantly expanded gene families.**

| **CAFE ID** | **Gene ID** | **pFAM ID** | **pFAM annotation** | **Blast nr descriptions** | **OM gene count** | **OB gene count** | **Octopus ancestral gene count** | **P-value** |
| --- | --- | --- | --- | --- | --- | --- | --- | --- |
| GENE1054 | Omin023884p | PF05380 | Pao retrotransposon peptidase | uncharacterized protein LOC100892047 | 98 | 0 | 24 | 3E-74 |
| GENE1111 | Omin006033p | PF13843 | Transposase IS4 | PiggyBac transposable element-derived protein 4 | 86 | 2 | 22 | 8E-64 |
| GENE1134 | Omin020631p | PF16064 | Domain of unknown function (DUF4806) | hypothetical protein CGI_10014324 | 78 | 0 | 18 | 2E-61 |
| GENE1128 | Omin020321p | PF13843 | Transposase IS4 | piggyBac transposable element-derived protein 4 | 74 | 0 | 18 | 8E-57 |
| GENE1165 | Omin000471p | PF14291 | Domain of unknown function (DUF4371) | zinc finger MYM-type protein 1 | 66 | 1 | 16 | 5E-51 |
| GENE1203 | Omin028576p | PF14291 | Domain of unknown function (DUF4371) | zinc finger MYM-type protein 1 | 58 | 1 | 15 | 2E-43 |
| GENE1323 | Omin008849p | PF00078 | Reverse transcriptase (RNA-dependent DNA polymerase) | polprotein | 41 | 1 | 11 | 1E-30 |
| GENE1231 | Omin028630p | PF00852 | Glycosyltransferase family 10 (fucosyltransferase) C-term | hypothetical protein OCBIM_22032667mg | 36 | 4 | 12 | 2E-23 |
| GENE1384 | Omin021959p | PF06083 | Interleukin-17 | Interleukin 17protein | 36 | 5 | 12 | 2E-23 |
| GENE1460 | Omin013080p | PF01498 | Transposase | Transposase domain containing protein | 33 | 1 | 9 | 6E-25 |
| GENE1514 | Omin016244p | PF01825 | GPCR proteolysis site, GPS, motif | GK12693 | 33 | 0 | 8 | 1E-26 |
| GENE1359 | Omin012952p | PF01825 | GPCR proteolysis site, GPS, motif | hypothetical protein OCBIM_22014869mg | 32 | 7 | 13 | 1E-17 |
| GENE1011 | Omin003748p | PF12774 | Hydrolytic ATP binding site of dynein motor region D1 | hypothetical protein OCBIM_22009963mg | 31 | 21 | 25 | 2E-03 |
| GENE1647 | Omin007822p | PF13358 | DDE superfamily endonuclease | uncharacterized protein LOC101846520 | 29 | 0 | 7 | 1E-23 |
| GENE1693 | Omin019999p | PF12874 | Zinc-finger of C2H2 type | hypothetical protein OCBIM_22013686mg | 28 | 1 | 8 | 8E-21 |
| GENE1692 | Omin026810p | PF14529 | Endonuclease-reverse transcriptase | hypothetical protein OCBIM_22022330mg | 28 | 1 | 8 | 8E-21 |
| GENE1606 | Omin022870p | PF05679 | Chondroitin N-acetylgalactosaminyltransferase | chondroitin sulfate synthase 1 | 27 | 4 | 9 | 4E-18 |
| GENE1746 | Omin029144p | PF00665 | Integrase core domain | unknown | 27 | 0 | 7 | 2E-21 |
| GENE1603 | Omin001040p | PF00078 | Reverse transcriptase (RNA-dependent DNA polymerase) | reverse transcriptase | 26 | 1 | 7 | 3E-20 |
| GENE1637 | Omin001834p | PF03184 | DDE superfamily endonuclease | tigger transposable element-derived protein 4 | 26 | 2 | 8 | 1E-18 |
| GENE1192 | Omin017279p | PF07690 | Major Facilitator Superfamily | Sialin | 22 | 20 | 20 | 1E-01 |
| GENE2272 | Omin028132p | PF00229 | TNF(Tumour Necrosis Factor) family | hypothetical protein | 21 | 1 | 6 | 4E-16 |
| GENE1802 | Omin013861p | PF00092 | von Willebrand factor type A domain | Collagen alpha-4(VI) chain | 21 | 2 | 7 | 2E-14 |
| GENE1512 | Omin014728p | PF05970 | PIF1-like helicase | ATP-dependent DNA helicase PIF1 | 21 | 13 | 15 | 2E-04 |
| GENE1034 | Omin024122p | PF00125 | Core histone H2A/H2B/H3/H4 | RecName: Full=Histone H2A | 20 | 7 | 13 | 1E-05 |
| GENE1239 | Omin011708p | PF00002 | 7 transmembrane receptor (Secretin family) | hypothetical protein OCBIM_22034326mg | 19 | 13 | 15 | 8E-03 |
| GENE2431 | Omin012389p | PF10324 | Serpentine type 7TM GPCR chemoreceptor Srw | P2Y purinoceptor 4 | 19 | 2 | 6 | 7E-14 |
| GENE1297 | Omin012114p | PF01411 | tRNA synthetases class II (A) | Alanyl-tRNA synthetase: cytoplasmic | 18 | 2 | 6 | 1E-12 |
| GENE1283 | Omin029077p | PF09172 | Domain of unknown function (DUF1943) | apolipophorins | 17 | 5 | 8 | 2E-08 |
| GENE2885 | Omin018594p | PF00515 | Tetratricopeptide repeat | hypothetical protein OCBIM_22039197mg | 17 | 2 | 6 | 1E-11 |

OM, *Octopus minor*; OB, *Octopus bimaculoides*

**Table S8. Example of top 30 CAFE significantly shrinked gene families.**

| **CAFE ID** | **Gene ID** | **pFAM ID** | **pFAM annotation** | **Blast nr descriptions** | **OM gene count** | **OB gene count** | **Octopus ancestral gene count** | **P-value** |
| --- | --- | --- | --- | --- | --- | --- | --- | --- |
| GENE1175 | Omin004782p | #N/A | #N/A | RNA-directed DNA polymerase from mobile element jockey | 25 | 40 | 28 | 1E-07 |
| GENE1164 | Omin022076p | PF14529 | Endonuclease-reverse transcriptase | hypothetical protein OCBIM_22004370mg | 6 | 32 | 13 | 1E-17 |
| GENE1801 | Omin002831p | PF12762 | ISXO2-like transposase domain | hypothetical protein OCBIM_22013099mg | 9 | 18 | 11 | 1E-05 |
| GENE1358 | Omin026033p | PF12762 | ISXO2-like transposase domain | hypothetical protein OCBIM_22021589mg | 2 | 39 | 11 | 2E-28 |
| GENE1410 | Omin002081p | PF05699 | hAT family C-terminal dimerisation region | hypothetical protein OCBIM_22037217mg | 6 | 13 | 8 | 1E-04 |
| GENE2267 | Omin003561p | PF05970 | PIF1-like helicase | uncharacterized protein LOC101240041 | 2 | 20 | 6 | 5E-15 |
| GENE1074 | Omin018025p | PF03953 | Tubulin C-terminal domain | beta-tubulin | 5 | 6 | 6 | 7E-01 |
| GENE1620 | Omin018346p | PF07732 | Multicopper oxidase | Laccase-2 | 4 | 6 | 5 | 1E-01 |
| GENE1066 | Omin010474p | PF07690 | Major Facilitator Superfamily | organic cation transporter protein | 3 | 4 | 4 | 7E-01 |
| GENE1262 | Omin023919p | PF03372 | Endonuclease/Exonuclease/phosphatase family | hypothetical protein Y032_0062g3370 | 3 | 6 | 4 | 3E-02 |
| GENE1118 | Omin006563p | PF12698 | ABC-2 family transporter protein | ATP-binding cassette sub-family A member 1 | 3 | 4 | 4 | 7E-01 |
| GENE8200 | Omin017282p | PF05970 | PIF1-like helicase | hypothetical protein OCBIM_22035338mg | 2 | 9 | 4 | 2E-05 |
| GENE1089 | Omin000303p | PF00209 | Sodium:neurotransmitter symporter family | Sodium- and chloride-dependent glycine transporter 2 | 3 | 4 | 4 | 7E-01 |
| GENE7296 | Omin027091p | PF12762 | ISXO2-like transposase domain | hypothetical protein TcasGA2_TC003734 | 2 | 8 | 3 | 4E-06 |
| GENE9885 | Omin024166p | PF05478 | Prominin | prominin 1 | 1 | 9 | 3 | 3E-07 |
| GENE1158 | Omin025499p | PF02736 | Myosin N-terminal SH3-like domain | myosin heavy chain isoform A | 2 | 3 | 3 | 6E-01 |
| GENE7299 | Omin006855p | PF01094 | Receptor family ligand binding region | hypothetical protein OCBIM_22030359mg | 1 | 7 | 3 | 5E-05 |
| GENE6991 | Omin010627p | PF05699 | hAT family C-terminal dimerisation region | general transcription factor II-I repeat domain-containing protein 2A | 1 | 9 | 3 | 3E-07 |
| GENE1195 | Omin018031p | PF00860 | Permease family | Solute carrier family 23 member 2 | 2 | 3 | 3 | 6E-01 |
| GENE9884 | Omin000788p | PF13843 | Transposase IS4 | piggyBac transposable element-derived protein 3 | 1 | 8 | 3 | 4E-06 |
| GENE1217 | Omin022888p | PF00209 | Sodium:neurotransmitter symporter family | taurine transporter | 2 | 3 | 3 | 6E-01 |
| GENE9891 | Omin029499p | PF00656 | Caspase domain | caspase-7 | 1 | 9 | 3 | 3E-07 |
| GENE1701 | Omin000701p | PF01549 | ShK domain-like | hypothetical protein OCBIM_22016617mg | 1 | 3 | 2 | 5E-02 |
| GENE1230 | Omin029346p | PF01582 | TIR domain | Tollreceptor 2 type-1 | 1 | 2 | 2 | 6E-01 |
| GENE1220 | Omin027474p | PF13087 | AAA domain | NFX1-type zinc finger-containing protein 1 | 1 | 2 | 2 | 6E-01 |
| GENE1176 | Omin009738p | PF00211 | Adenylate and Guanylate cyclase catalytic domain | atrial natriuretic peptide receptor 1 | 1 | 2 | 2 | 6E-01 |
| GENE12620 | Omin025894p | PF00083 | Sugar (and other) transporter | Organic cation transporter protein | 1 | 6 | 2 | 2E-05 |
| GENE11615 | Omin009045p | PF01753 | MYND finger | hypothetical protein OCBIM_22032555mg | 1 | 5 | 2 | 3E-04 |
| GENE1627 | Omin029134p | PF00008 | EGF-like domain | IgGFc-binding protein | 1 | 2 | 2 | 6E-01 |
| GENE12606 | Omin010680p | PF13843 | Transposase IS4 | piggyBac transposable element-derived protein 4 | 1 | 6 | 2 | 2E-05 |

OM, *Octopus minor*; OB, *Octopus bimaculoides*

**Table S9. Top 30 expanded Pfam domains.**

| Pfam ID | Descriptions | OM | OB | LG | PF | CG | LA | CT | HR | CE | DM | DP | SP | MM | HS |
| --- | --- | --- | --- | --- | --- | --- | --- | --- | --- | --- | --- | --- | --- | --- | --- |
| PF00028 | Cadherin domain | 323 | 224 | 57 | 69 | 66 | 46 | 62 | 79 | 12 | 14 | 18 | 25 | 118 | 527 |
| PF14291 | Domain of unknown function (DUF4371) | 301 | 93 | 0 | 0 | 19 | 16 | 4 | 9 | 0 | 0 | 0 | 13 | 5 | 3 |
| PF13843 | Transposase IS4 | 171 | 71 | 3 | 1 | 6 | 15 | 7 | 4 | 0 | 2 | 0 | 6 | 4 | 0 |
| PF05699 | hAT family C-terminal dimerisation region | 118 | 25 | 2 | 2 | 29 | 20 | 63 | 187 | 8 | 1 | 40 | 33 | 6 | 4 |
| PF00188 | Cysteine-rich secretory protein family | 100 | 19 | 17 | 27 | 30 | 29 | 19 | 22 | 34 | 31 | 19 | 24 | 13 | 0 |
| PF06083 | Interleukin-17 | 72 | 37 | 10 | 12 | 8 | 4 | 11 | 1 | 3 | 1 | 2 | 11 | 6 | 0 |
| PF03074 | Glutamate-cysteine ligase | 46 | 11 | 1 | 1 | 0 | 6 | 2 | 1 | 1 | 1 | 1 | 1 | 1 | 0 |
| PF05679 | Chondroitin N-acetylgalactosaminyltransferase | 39 | 13 | 7 | 2 | 3 | 8 | 6 | 0 | 2 | 3 | 5 | 3 | 8 | 6 |
| PF16211 | C-terminus of histone H2A | 38 | 17 | 19 | 10 | 5 | 8 | 27 | 5 | 19 | 21 | 24 | 24 | 21 | 0 |
| PF01498 | Transposase | 37 | 4 | 0 | 0 | 7 | 3 | 5 | 0 | 0 | 1 | 13 | 2 | 0 | 0 |
| PF00305 | Lipoxygenase | 31 | 9 | 4 | 13 | 2 | 7 | 4 | 3 | 0 | 0 | 0 | 20 | 6 | 4 |
| PF01510 | N-acetylmuramoyl-L-alanine amidase | 28 | 3 | 6 | 8 | 9 | 13 | 6 | 0 | 0 | 10 | 0 | 4 | 4 | 0 |
| PF00264 | Common central domain of tyrosinase | 27 | 16 | 3 | 25 | 39 | 7 | 3 | 1 | 6 | 0 | 0 | 1 | 3 | 0 |
| PF00233 | 3'5'-cyclic nucleotide phosphodiesterase | 23 | 21 | 14 | 13 | 16 | 15 | 11 | 17 | 6 | 6 | 12 | 17 | 23 | 16 |
| PF16064 | Domain of unknown function (DUF4806) | 22 | 0 | 0 | 6 | 2 | 2 | 12 | 0 | 0 | 2 | 21 | 1 | 0 | 0 |
| PF00110 | wnt family | 22 | 17 | 12 | 12 | 11 | 17 | 12 | 18 | 5 | 7 | 12 | 13 | 22 | 0 |
| PF13358 | DDE superfamily endonuclease | 20 | 6 | 2 | 3 | 20 | 0 | 0 | 0 | 1 | 1 | 13 | 10 | 0 | 0 |
| PF00109 | Beta-ketoacyl synthase, N-terminal domain | 17 | 4 | 10 | 7 | 11 | 13 | 8 | 2 | 4 | 4 | 1 | 10 | 2 | 1 |
| PF00086 | Thyroglobulin type-1 repeat | 17 | 12 | 4 | 6 | 10 | 12 | 3 | 5 | 5 | 3 | 7 | 10 | 14 | 12 |
| PF03221 | Tc5 transposase DNA-binding domain | 17 | 3 | 5 | 0 | 15 | 7 | 15 | 4 | 4 | 5 | 7 | 10 | 14 | 1 |
| PF09612 | Bacterial protein of unknown function (HtrL_YibB) | 16 | 9 | 3 | 13 | 6 | 5 | 3 | 2 | 1 | 0 | 0 | 0 | 0 | 0 |
| PF00550 | Phosphopantetheine attachment site | 15 | 6 | 5 | 6 | 10 | 15 | 12 | 3 | 6 | 6 | 2 | 4 | 5 | 3 |
| PF09607 | Brinker DNA-binding domain | 14 | 4 | 1 | 0 | 1 | 0 | 0 | 0 | 2 | 1 | 0 | 1 | 1 | 0 |
| PF02801 | Beta-ketoacyl synthase, C-terminal domain | 14 | 4 | 9 | 5 | 10 | 10 | 9 | 2 | 3 | 4 | 1 | 8 | 2 | 1 |
| PF00292 | 'Paired box' domain | 14 | 5 | 7 | 9 | 11 | 8 | 5 | 10 | 10 | 10 | 13 | 7 | 9 | 0 |
| PF08450 | SMP-30/Gluconolaconase/LRE-like region | 12 | 9 | 4 | 10 | 8 | 4 | 4 | 0 | 1 | 2 | 0 | 7 | 1 | 0 |
| PF01966 | HD domain | 12 | 3 | 3 | 6 | 3 | 1 | 3 | 3 | 1 | 1 | 0 | 16 | 1 | 0 |
| PF00210 | Ferritin-like domain | 11 | 6 | 5 | 5 | 4 | 7 | 4 | 4 | 2 | 3 | 9 | 2 | 4 | 0 |
| PF02463 | RecF/RecN/SMC N terminal domain | 11 | 4 | 5 | 5 | 4 | 8 | 6 | 3 | 8 | 6 | 11 | 9 | 5 | 6 |
| PF02272 | DHHA1 domain | 10 | 1 | 1 | 1 | 1 | 1 | 1 | 1 | 1 | 1 | 1 | 2 | 0 | 1 |

OM, *Octopus minor*; OB, *Octopus bimaculoides*; LG, *Lottia gigantean*; CG, *Crassostrea gigas*; PF, *Pinctada fucata*; LA, *Lingula anatina*; CT, *Capitella teleta*; HR, *Helobdella robusta*; CE, *Caenorhabditis elegans*; DM, *Drosophila melanogaster*; DP, *Daphnia pulex*; SP, *Strongylocentrotus purpuratus*; MM, *Mus musculus*; HS, *Homo sapiens*

**Table S10. Top 30 expanded EggNOG domains.**

| Eggnog ID | Type | Descriptions | OM | OB | LG | PF | CG | LA | CT | HR | CE | DM | DP | SP | MM | HS |
| --- | --- | --- | --- | --- | --- | --- | --- | --- | --- | --- | --- | --- | --- | --- | --- | --- |
| ENOG410V8KB | U | Protocadherin | 268 | 180 | 17 | 19 | 16 | 0 | 31 | 49 | 0 | 0 | 0 | 1 | 4 | 5 |
| ENOG410VH5S | EPT | piggyBac transposable element derived 4 | 156 | 25 | 1 | 3 | 0 | 8 | 4 | 0 | 0 | 0 | 0 | 4 | 0 | 1 |
| ENOG410VFBG | S | zinc finger, MYM-type | 149 | 8 | 0 | 2 | 0 | 0 | 2 | 6 | 0 | 0 | 0 | 4 | 1 | 1 |
| ENOG410VK1E | S | Inherit from opiNOG: protein Hydra magnipapillata | 137 | 113 | 0 | 2 | 0 | 2 | 6 | 19 | 1 | 0 | 0 | 3 | 0 | 0 |
| ENOG410V8N0 | H | glutamate--cysteine ligase catalytic subunit | 45 | 12 | 1 | 0 | 1 | 7 | 2 | 1 | 1 | 1 | 1 | 1 | 1 | 1 |
| ENOG410V6S6 | T | G- protein-coupled receptor | 44 | 27 | 4 | 4 | 4 | 4 | 3 | 7 | 1 | 2 | 0 | 9 | 3 | 3 |
| ENOG410VI9I | B | Core component of nucleosome | 38 | 23 | 12 | 1 | 8 | 0 | 16 | 10 | 16 | 23 | 16 | 16 | 9 | 12 |
| ENOG410VD6J | S | interleukin 17c | 38 | 15 | 7 | 4 | 2 | 0 | 0 | 0 | 0 | 0 | 0 | 1 | 1 | 1 |
| ENOG410V7PZ | S | Arachidonate | 36 | 0 | 4 | 3 | 13 | 10 | 4 | 3 | 0 | 0 | 0 | 6 | 5 | 6 |
| ENOG410V4I9 | M | peptidoglycan recognition protein | 35 | 4 | 1 | 2 | 1 | 4 | 2 | 0 | 0 | 2 | 0 | 0 | 3 | 3 |
| ENOG410VD39 | G | Alpha 1,3 fucosyltransferase | 35 | 6 | 6 | 8 | 7 | 7 | 23 | 4 | 32 | 1 | 2 | 24 | 2 | 5 |
| ENOG410V97R | G | Chondroitin sulfate synthase | 34 | 10 | 7 | 2 | 1 | 5 | 2 | 0 | 1 | 1 | 1 | 1 | 2 | 2 |
| ENOG410VI9V | B | Histone H2A | 34 | 18 | 20 | 3 | 10 | 5 | 21 | 10 | 19 | 3 | 17 | 2 | 6 | 7 |
| ENOG410V4YE | Z | Dynein, axonemal, heavy chain | 30 | 14 | 7 | 12 | 14 | 17 | 9 | 14 | 1 | 5 | 0 | 6 | 6 | 5 |
| ENOG410WI2Q | TV | peptidase inhibitor activity | 27 | 18 | 3 | 2 | 5 | 1 | 13 | 3 | 1 | 1 | 2 | 1 | 0 | 0 |
| ENOG410WCUR | S | Integrase core domain | 26 | 1 | 1 | 6 | 2 | 12 | 13 | 1 | 0 | 0 | 0 | 2 | 0 | 0 |
| ENOG410V7QW | O | ankyrin repeat domain | 24 | 10 | 6 | 8 | 19 | 12 | 14 | 2 | 4 | 0 | 0 | 17 | 3 | 2 |
| ENOG410V5FA | VW | cysteine-rich secretory protein LCCL domain containing | 18 | 2 | 0 | 0 | 4 | 1 | 0 | 0 | 1 | 1 | 4 | 1 | 2 | 2 |
| ENOG410WHV6 | I | Domain of Unknown Function (DUF1081) | 18 | 12 | 3 | 2 | 9 | 2 | 2 | 1 | 2 | 1 | 0 | 1 | 0 | 1 |
| ENOG410V74K | A | SAM domain and HD | 18 | 3 | 3 | 3 | 8 | 3 | 3 | 3 | 0 | 1 | 1 | 8 | 1 | 1 |
| ENOG410V699 | BD | tigger transposable element derived | 17 | 1 | 0 | 9 | 0 | 0 | 6 | 5 | 5 | 0 | 0 | 1 | 1 | 2 |
| ENOG410V55F | T | ACEtylcholinesterase | 16 | 12 | 5 | 6 | 7 | 8 | 10 | 5 | 2 | 1 | 4 | 1 | 2 | 2 |
| ENOG410V4Q9 | Z | Dynein, axonemal, heavy chain | 16 | 6 | 5 | 9 | 5 | 11 | 3 | 11 | 1 | 2 | 0 | 5 | 3 | 3 |
| ENOG410V5BP | J | alanyl-tRNA synthetase | 16 | 0 | 3 | 5 | 2 | 3 | 2 | 2 | 2 | 2 | 1 | 2 | 2 | 2 |
| ENOG410VIH7 | G | ec 2.4.1.62 | 15 | 5 | 9 | 2 | 2 | 2 | 7 | 4 | 1 | 1 | 2 | 1 | 0 | 0 |
| ENOG410V9ZG | BD | pogo transposable element with KRAB domain | 15 | 5 | 3 | 0 | 0 | 2 | 1 | 11 | 0 | 0 | 1 | 2 | 1 | 1 |
| ENOG410VEC3 | D | Structural maintenance of chromosomes | 15 | 1 | 1 | 1 | 2 | 2 | 1 | 1 | 2 | 1 | 1 | 1 | 1 | 1 |
| ENOG410VEVI | G | solute carrier family 16, member 14 (monocarboxylic acid transporter 14) | 14 | 7 | 4 | 12 | 4 | 1 | 10 | 2 | 0 | 0 | 2 | 1 | 1 | 0 |
| ENOG410VQH0 | S | Bacterial protein of unknown function (HtrL_YibB) | 14 | 8 | 3 | 5 | 12 | 3 | 3 | 2 | 0 | 0 | 1 | 0 | 0 | 0 |
| ENOG410WI6V | T | Inherit from KOG: protein tyrosine phosphatase receptor type | 14 | 10 | 1 | 12 | 7 | 3 | 4 | 2 | 0 | 0 | 0 | 0 | 0 | 0 |

OM, *Octopus minor*; OB, *Octopus bimaculoides*; LG, *Lottia gigantean*; CG, *Crassostrea gigas*; PF, *Pinctada fucata*; LA, *Lingula anatina*; CT, *Capitella teleta*; HR, *Helobdella robusta*; CE, *Caenorhabditis elegans*; DM, *Drosophila melanogaster*; DP, *Daphnia pulex*; SP, *Strongylocentrotus purpuratus*; MM, *Mus musculus*; HS, *Homo sapiens*

**Table S11. Statistics of repeat analysis of the *O. minor* genome.**

|  | ***O. minor*** | | | ***O. bimaculoides*** | | |
| --- | --- | --- | --- | --- | --- | --- |
|  | Count | Total length (bp) | % genome | Count | Total length (bp) | % genome |
| DNA Transposon | 1,224,219 | 648,926,303 | 12.75 | 696,864 | 272,928,679 | 11.67 |
| LINE | 939,053 | 527,037,971 | 10.35 | 462,986 | 207,216,782 | 8.86 |
| SINE | 1,540 | 474,165 | 0.01 | 115,169 | 43,709,659 | 1.87 |
| LTR Retrotransposon | 275,498 | 114,225,073 | 2.24 | 213,966 | 68,596,656 | 2.93 |
| Satellite | 71,572 | 29,324,204 | 0.58 | 36,649 | 12,563,346 | 0.54 |
| RC-Helitron | 121,101 | 189,161,340 | 3.72 | 43,735 | 15,496,549 | 0.66 |
| Unclassified | 8,342 | 2,257,147 | 0.04 | 9,445 | 2,851,162 | 0.12 |
| Simple Repeat | 8,904,555 | 749,744,345 | 14.73 | 2,307,355 | 202,517,198 | 8.66 |
| snRNA | 949 | 503,393 | 0.01 | 492 | 39,598 | 0 |
| RNA (5S_DM) | 496 | 228,944 | 0 | 364 | 180,724 | 0.01 |
| **Total** | **11,547,325** | **2,261,882,885** | **44.43** | **3,887,025** | **826,100,353** | **35.33** |

**Table S12. Classifications and frequencies of transposable elements and other repeats.**

| **Classes** | **Count** | **Bases masked (bp)** | **% repeat** |
| --- | --- | --- | --- |
| **DNA transposons** |  |  |  |
| TcMar | 347,113 | 422,507,413 | 8.30 |
| hAT | 339,610 | 272,282,913 | 5.35 |
| BREP1 | 286,060 | 188,045,526 | 3.69 |
| CMC | 270,691 | 180,865,551 | 3.55 |
| rnd | 136,523 | 153,966,258 | 3.02 |
| MULE | 78,779 | 76,985,540 | 1.51 |
| Novosib | 95,251 | 50,033,218 | 0.98 |
| Sola | 95,366 | 38,912,101 | 0.76 |
| Ginger | 64,083 | 23,243,243 | 0.46 |
| Maverick | 57,750 | 18,271,614 | 0.36 |
| **Retrotransposons** |  |  |  |
| **LINE** |  |  |  |
| L1-Tx1 | 189,077 | 1,269,800,359 | 24.95 |
| Dong-R4 | 479,154 | 1,260,915,183 | 24.77 |
| Penelope | 401,054 | 837,326,060 | 16.45 |
| RTE-BovB | 660,445 | 665,190,664 | 13.07 |
| CR1 | 132,501 | 463,884,204 | 9.11 |
| L2 | 372,618 | 281,066,684 | 5.52 |
| **LTR** |  |  |  |
| Gypsy | 250,185 | 288,182,298 | 5.66 |
| ERV | 87,564 | 26,776,666 | 0.53 |
| Copia | 11,567 | 5,238,650 | 0.10 |
| Ngaro | 7,102 | 1,990,679 | 0.04 |
| DIRS | 4,258 | 1,304,311 | 0.03 |
| Pao | 2,397 | 691,357 | 0.01 |
| **SINE** |  |  |  |
| RTE | 460 | 23,275,005 | 0.46 |
| MIR | 5,041 | 1,015,899 | 0.02 |
| Mermaid | 2 | 712,649 | 0.01 |
| B2 | 898 | 152,687 | 0.00 |
| B4 | 164 | 36,196 | 0.00 |
| Low complexity | 482,843 | 12,628,685 | 0.25 |
| Satellite | 71,572 | 29,324,204 | 0.58 |
| Helitron | 121,101 | 189,161,340 | 3.72 |
| Simple Repeat | 8,904,555 | 749,744,345 | 14.73 |

**Table S13. Classifications and frequencies of simple repeats.**

| **Simple repeat** | **Count** | **Total length (bp)** |
| --- | --- | --- |
| (TA)n | 1,912,052 | 170,327,543 |
| (AT)n | 1,845,607 | 158,149,821 |
| (TG)n | 331,273 | 14,136,386 |
| (AC)n | 286,907 | 10,452,694 |
| (TATA)n | 50,090 | 10,180,211 |
| (TATG)n | 89,927 | 9,598,768 |
| (ATAT)n | 51,196 | 9,433,815 |
| (ATG)n | 50,559 | 8,004,354 |
| (CA)n | 171,755 | 7,674,832 |
| (CAT)n | 36,585 | 7,420,350 |
