# Supplementary material for: The genome of common long-arm octopus Octopus minor
Source: Gigascience. 2018 Sep 25;7(11):giy119. doi: 10.1093/gigascience/giy119 (PMC6279123; doi:10.1093/gigascience/giy119)
Supplement: Supplemental Files [file giy119_supplemental_files.zip › Supplementary text_commands.docx]

1. Genome sequencing and annotation
   1. FALCON-UNZIP ver. 0.4

**#step1**

*$fc_run.py input.cfg*

input.cfg

| [General]  input_fofn = input.fofn  input_type = raw  length_cutoff = 10000  length_cutoff_pr = 8000  sge_option_da = -pe orte 4 -q utl.q  sge_option_la = -pe orte 2 -q utl.q  sge_option_pda = -pe orte 8 -q utl.q  sge_option_pla = -pe orte 8 -q utl.q  sge_option_fc = -pe orte 24 -q utl.q  sge_option_cns = -pe orte 8 -q utl.q  pa_concurrent_jobs = 96  cns_concurrent_jobs = 96  ovlp_concurrent_jobs = 96  pa_HPCdaligner_option = -b -v -dal128 -t16 -M32 -e.70 -l4800 -s100 -k18 -h480 -w8 -H15000  ovlp_HPCdaligner_option = -v -dal128 -t32 -M32 -h240 -e.96 -l500 -s1000 -k18 -h240 -w5  pa_DBsplit_option = -a -x500 -s400  ovlp_DBsplit_option = -x500 -s400  falcon_sense_option = --output_multi --min_idt 0.70 --min_cov 4 --max_n_read 200 --n_core 24  overlap_filtering_setting = --max_diff 60 --max_cov 60 --min_cov 2 --n_core 24 |
| --- |

**#step2**

*$fc_unzip.py fc_unzip.cfg*

fc_unzip.cfg

| [General]  job_type = SGE  [Unzip]  input_fofn=input.fofn  input_bam_fofn= input.bam.fofn  smrt_bin=/home/smrtanalysis/smrtlink/smrtcmds/bin/  jobqueue = utl.q  sge_phasing= -pe orte 12 -q %(jobqueue)s  sge_quiver= -pe orte 12 -q %(jobqueue)s  sge_track_reads= -pe orte 12 -q %(jobqueue)s  sge_blasr_aln= -pe orte 24 -q %(jobqueue)s  sge_hasm= -pe orte 32 -q %(jobqueue)s  unzip_concurrent_jobs = 64  quiver_concurrent_jobs = 64 |
| --- |

**#step3**

*$fc_quiver.py fc_unzip.cfg*

- 1. Tofu

*$tofu_wrap.py --nfl_fa $all_isoseq_nfl_fasta -d IsoSeq_Results --ccs_fofn $all_reads_of_insert_fofn --bas_fofn $all_input_fofn --gmap_db /dlabs/genome --gmap_name hg19 --quiver --use_sge --sge_queue $queue --max_sge_jobs 40 --unique_id 300 --blasr_nproc 24 --quiver_nproc 8 --hq_isoforms_fa all_quivered_hq.fasta --lq_isoforms_fa all_quivered_lq.fasta $all_isoseq_flnc_fasta final.consensus.fa*

- 1. Chemera removal (In-house script)

*#!/bin/bash*

*fasta=$1*

*RNAfold --noPS < $fasta > $fasta.rnafold.out*

*paste - - - $fasta.rnafold.out | grep ‘({200}’ | awk ‘{print $1;}’ > seq.id*

*fastaindex –f $fasta –i $fasta.idx*

*while read line; do fastafetch –f $fasta –i $fasta.idx –q $line >> $fasta.remove.fasta; done < seq.id*

- 1. BUSCO ver. 1.22
     1. Eukaryotes DB

*$ Python3 BUSCO_v1.22.py -o <SAMPLE_ID> -in <assembly.fasta> -l eukaryota -m genome -c 32 –long*

- - 1. Metazoa DB

*$ Python3 BUSCO_v1.22.py -o <SAMPLE_ID> -in <assembly.fasta> -l metazoa -m genome -c 32 --long*

- 1. RepeatMasker ver. 4.0.7

**#step 1**

*$perl BuildDatabase -name OminorRep ../Ominor-genome.fasta*

**#step 2**

*$perl RepeatModeler -engine ncbi -pa 80 -database OminorRep*

**#step 3**

*$RepeatMasker -lib consensi.fa.classified ../Ominor-genome.fasta -pa 80*

- 1. MAKER

**#step 1- 1^st^ MAKER training**

$maker –CTL

maker_opts.ctl

| #-----Genome (these are always required)  genome= Ominor-genome.fasta #genome sequence (fasta file or fasta embeded in GFF3 file)  organism_type=eukaryotic #eukaryotic or prokaryotic. Default is eukaryotic  #-----Re-annotation Using MAKER Derived GFF3  maker_gff= #MAKER derived GFF3 file  est_pass=0 #use ESTs in maker_gff: 1 = yes, 0 = no  altest_pass=0 #use alternate organism ESTs in maker_gff: 1 = yes, 0 = no  protein_pass=0 #use protein alignments in maker_gff: 1 = yes, 0 = no  rm_pass=0 #use repeats in maker_gff: 1 = yes, 0 = no  model_pass=0 #use gene models in maker_gff: 1 = yes, 0 = no  pred_pass=0 #use ab-initio predictions in maker_gff: 1 = yes, 0 = no  other_pass=0 #passthrough anyything else in maker_gff: 1 = yes, 0 = no  #-----EST Evidence (for best results provide a file for at least one)  est= Isoseq-unmap-add-161226.fasta #set of ESTs or assembled mRNA-seq in fasta format  altest= #EST/cDNA sequence file in fasta format from an alternate organism  est_gff= #aligned ESTs or mRNA-seq from an external GFF3 file  altest_gff= #aligned ESTs from a closly relate species in GFF3 format  #-----Protein Homology Evidence (for best results provide a file for at least one)  protein= Ref-oct-AA.fasta #protein sequence file in fasta format (i.e. from mutiple oransisms)  protein_gff= #aligned protein homology evidence from an external GFF3 file  #-----Repeat Masking (leave values blank to skip repeat masking)  model_org=all #select a model organism for RepBase masking in RepeatMasker  rmlib= om.falcon.3.quiver.rplib.fasta #provide an organism specific repeat library in fasta format for RepeatMasker  repeat_protein= te_proteins.fasta #provide a fasta file of transposable element proteins for RepeatRunner  rm_gff= #pre-identified repeat elements from an external GFF3 file  prok_rm=0 #forces MAKER to repeatmask prokaryotes (no reason to change this), 1 = yes, 0 = no  softmask=1 #use soft-masking rather than hard-masking in BLAST (i.e. seg and dust filtering)  #-----Gene Prediction  snaphmm= /snapHMM/minimal.hmm #SNAP HMM filertemia  gmhmm= #GeneMark HMM file  augustus_species= #Augustus gene prediction species model  fgenesh_par_file= #FGENESH parameter file  pred_gff= #ab-initio predictions from an external GFF3 file  model_gff= #annotated gene models from an external GFF3 file (annotation pass-through)  est2genome=1 #infer gene predictions directly from ESTs, 1 = yes, 0 = no  protein2genome=1 #infer predictions from protein homology, 1 = yes, 0 = no  unmask=0 #also run ab-initio prediction programs on unmasked sequence, 1 = yes, 0 = no  #-----Other Annotation Feature Types (features MAKER doesn't recognize)  other_gff= #extra features to pass-through to final MAKER generated GFF3 file  #-----External Application Behavior Options  alt_peptide=C #amino acid used to replace non-standard amino acids in BLAST databases  cpus=1 #max number of cpus to use in BLAST and RepeatMasker (not for MPI, leave 1 when using MPI)  #-----MAKER Behavior Options  max_dna_len=1000000 #length for dividing up contigs into chunks (increases/decreases memory usage)  min_contig=1 #skip genome contigs below this length (under 10kb are often useless)  pred_flank=200 #flank for extending evidence clusters sent to gene predictors  pred_stats=0 #report AED and QI statistics for all predictions as well as models  AED_threshold=1 #Maximum Annotation Edit Distance allowed (bound by 0 and 1)  min_protein=0 #require at least this many amino acids in predicted proteins  alt_splice=0 #Take extra steps to try and find alternative splicing, 1 = yes, 0 = no  always_complete=0 #extra steps to force start and stop codons, 1 = yes, 0 = no  map_forward=0 #map names and attributes forward from old GFF3 genes, 1 = yes, 0 = no  keep_preds=0 #Concordance threshold to add unsupported gene prediction (bound by 0 and 1)  split_hit=100000 #length for the splitting of hits (expected max intron size for evidence alignments)  single_exon=0 #consider single exon EST evidence when generating annotations, 1 = yes, 0 = no  single_length=250 #min length required for single exon ESTs if 'single_exon is enabled'  correct_est_fusion=0 #limits use of ESTs in annotation to avoid fusion genes  tries=2 #number of times to try a contig if there is a failure for some reason  clean_try=0 #remove all data from previous run before retrying, 1 = yes, 0 = no  clean_up=0 #removes theVoid directory with individual analysis files, 1 = yes, 0 = no  TMP= #specify a directory other than the system default temporary directory for temporary files |
| --- |

*$mpiexec -n 80 maker </dev/null >maker.log & > maker1.out*

*$gff3_merge -d ../Ominor-genome.maker.output/ Ominor-genome_master_datastore_index.log –o Ominor-genome.gff3*

*$ maker2zff Ominor-genome.gff3*

*$fathom -categorize 1000 genome.ann genome.dna*

*$fathom -export 1000 -plus uni.ann uni.dna*

*$forge export.ann export.dna*

*$hmm-assembler.pl Pult . > Pult.hmm*

**#step 2 – 2^nd^ MAKER training**

maker_opts.ctl – modify gene prediction part as follows

| #-----Gene Prediction  snaphmm= Ominor-genome.maker.output/snap1/Pult.hmm #SNAP HMM filertemia  gmhmm= #GeneMark HMM file  augustus_species= #Augustus gene prediction species model  fgenesh_par_file= #FGENESH parameter file  pred_gff= #ab-initio predictions from an external GFF3 file  model_gff= #annotated gene models from an external GFF3 file (annotation pass-through)  est2genome=0 #infer gene predictions directly from ESTs, 1 = yes, 0 = no  protein2genome=0 #infer predictions from protein homology, 1 = yes, 0 = no  unmask=0 #also run ab-initio prediction programs on unmasked sequence, 1 = yes, 0 = no |
| --- |

*$mpiexec -n 80 maker </dev/null >maker.log & > maker2.out*

*$gff3_merge -d ../Ominor-genome.maker.output/ Ominor-genome_master_datastore_index.log –o Ominor-genome.gff3*

*$maker2zff Ominor-genome.gff3*

*$fathom -categorize 1000 genome.ann genome.dna*

*$fathom -export 1000 -plus uni.ann uni.dna*

*$forge export.ann export.dna*

*$hmm-assembler.pl Pult . > Pult.hmm*

**#step 3 – 3^rd^ MAKER training**

maker_opts.ctl– modify gene prediction part as follows

| #-----Gene Prediction  snaphmm= Ominor-genome.maker.output/snap2/Pult.hmm #SNAP HMM filertemia  gmhmm= #GeneMark HMM file  augustus_species= #Augustus gene prediction species model  fgenesh_par_file= #FGENESH parameter file  pred_gff= #ab-initio predictions from an external GFF3 file  model_gff= #annotated gene models from an external GFF3 file (annotation pass-through)  est2genome=0 #infer gene predictions directly from ESTs, 1 = yes, 0 = no  protein2genome=0 #infer predictions from protein homology, 1 = yes, 0 = no  unmask=0 #also run ab-initio prediction programs on unmasked sequence, 1 = yes, 0 = no |
| --- |

*$mpiexec -n 80 maker </dev/null >maker.log & > maker3.out*

*$gff3_merge –d ../Ominor-genome.maker.output/Ominor-genome _master_datastore_index.log -o Ominor.gff3*

*$fasta_merge -d ../Ominor-genome.maker.output/Ominor-genome _master_datastore_index.log -o Ominor*

1. Comparative genomic analyses and duplicated genes
   1. OrthomMCL ver. 2.0.9

**# step 1**

*$service mysqld start*

*$mysql*

*mysql> show databases;*

*mysql> drop database orthomcl*

*mysql> CREATE DATABASE orthomcl;*

*mysql> GRANT SELECT,INSERT,UPDATE,DELETE,CREATE VIEW,CREATE, INDEX, DROP on orthomcl.* TO orthomcl@localhost;mysql> set password for orthomcl@localhost = password('123123');*

**# step2**

*$orthomclInstallSchema ./orthomcl.config.template*

orthomcl.config.template

| $ this config assumes a mysql database named 'orthomcl'. adjust according  $ to your situation.  dbVendor=mysql  dbConnectString=dbi:mysql:orthomcl  dbLogin=orthomcl  dbPassword=123123  similarSequencesTable=SimilarSequences  orthologTable=Ortholog  inParalogTable=InParalog  coOrthologTable=CoOrtholog  interTaxonMatchView=InterTaxonMatch  percentMatchCutoff=50  evalueExponentCutoff=-4  oracleIndexTblSpc=NONE |
| --- |

**# step 3**

*$orthomclAdjustFasta taxon_ID taxon.fa '.'*

**# step 4**

move all fasta files into the directory named FASTA

**# step 5**

*$orthomclFilterFasta ./FASTA 10 20*

**# step 6**

*$formatdb -i goodProteins.fasta -p F*

**# step 7**

*$blastall -p tblastx -d ./goodProteins.fasta -i ./goodProteins.fasta -m 8 -o tblastX*

**# step 8**

*$orthomclBlastParser tblastX ./FASTA/ >>SimilarSequence.txt*

**# step 9**

*$orthomclLoadBlast ./orthomcl.config.template ./SimilarSequence.txt*

**# step 10**

*$orthomclPairs ./orthomcl.config.template ./orthmcl_pairs.log cleanup=no*

**# step 11**

*$orthomclDumpPairsFiles ./orthomcl.config.template*

**# step 12**

*$mcl ./mclInput --abc -I 1.5 -o ./mclOutput*

**# step 13**

*$orthomclMclToGroups GENE 1000 < mclOutput > groups.txt*

- 1. Pfam domain assignments

*$ interproscan.sh -i OB.prot.fasta -f tsv -iprlookup -goterms -pa -appl Pfam -o OB.Pfam*

- 1. PRANK ver. 14063

*$prank -DNA -codon -o=gene_no.prank -d= gene_no.fasta +F*

- 1. Gblocks ver. 0.91b

*$ Gblocks gene_no.prank.best.fas gene_no.result.best.fas -t=c -e=-gb1 -b4=5 -d=y > gblock.log*

- 1. RAxML ver. 8.2.4

Windows GUI version 1,000 bootstrap

- 1. CAFÉ ver. 4.0

**# step 1**

prepare tab delimited CAFÉ input data using orthomcl output file groups.txt

octo_CAFE_input.txt

| Description ID CE CG CT DM DP HR HS LA LG MM OB OM PF SP  GENE23890 0 0 1 0 0 0 1 0 0 1 0 0 0 0  GENE14225 0 1 0 0 0 0 0 1 1 0 1 1 1 0  GENE35719 0 0 0 1 1 0 0 0 0 0 0 0 0 0  GENE28840 0 0 0 0 0 0 0 2 0 0 0 0 0 0  GENE17155 0 0 0 1 3 0 0 0 0 0 0 0 0 0  GENE24994 2 0 0 0 0 0 0 0 0  .  .  . |
| --- |

**# step 2**

*$python cafetutorial_clade_and_size_filter.py –i octo_CAFE_input.txt*

**# step 3**

$cafe

*café> load -i ./filtered_octo_CAFE_input.txt -t 10 -l ./logfile.txt -p 0.05*

*café>tree (((((((OB:38,OM:38):336,LG:374):76,(CG:328,PF:328):123):84,LA:534):69,(CT:506,HR:506):96):65,(CE:605,(DM:508,DP:508):98):62):0.000000000000000000001,(SP:574,(HS:89,MM:89):485):93);*

*café>lambda -s -t (((((((1,1)1,1)1,(1,1)1)1,1)1,(2,2)2)2,(2,(2,2)2)2)2,(2,(2,2)2)2);*

*café>report OCTO_resultfile*

**# step 4**

*$python ./cafetutorial_report_analysis.py -i OCTO_resultfile -o summary*

- 1. yn00 within PAML package ver. 4.7a

*$ yn00*

yn00.ctl

| seqfile = infile * sequence data file name  outfile = yn * main result file  verbose = 0 * 1: detailed output (list sequences), 0: concise output  icode = 0 * 0:universal code; 1:mammalian mt; 2-10:see below  weighting = 0 * weighting pathways between codons (0/1)?  commonf3x4 = 0 * use one set of codon freqs for all pairs (0/1)?  * ndata = 1  * Genetic codes: 0:universal, 1:mammalian mt., 2:yeast mt., 3:mold mt.,  * 4: invertebrate mt., 5: ciliate nuclear, 6: echinoderm mt.,  * 7: euplotid mt., 8: alternative yeast nu. 9: ascidian mt.,  * 10: blepharisma nu.  * These codes correspond to transl_table 1 to 11 of GENEBANK. |
| --- |
