# Supplementary material for: The genome of common long-arm octopus Octopus minor
Source: Gigascience. 2018 Sep 25;7(11):giy119. doi: 10.1093/gigascience/giy119 (PMC6279123; doi:10.1093/gigascience/giy119)
Supplement: Reviewer_1_Report_(Original_Submission) -- Joseph F. Ryan — 6/18/2018 Reviewed [file giy119_reviewer_1_report_(original_submission).pdf]

Reviewer Report

Title: **The genome of common long-arm octopus Octopus minor**

Version: **Original Submission** **Date:** 6/18/2018

Reviewer name: **Joseph F. Ryan**

Reviewer Comments to Author:

This is a nicely written data note describing a very interesting and important genomic resource, the genome of Octopus minor. The data and assembly seem reasonable.

1. Page 3, Line 24: "As advanced invertebrates,"

> "Advanced" implies that these animals have been evolving longer than other invertebrates. This is not true and this sentence would be improved if this phrase was removed.

2. Page 4, Line 78: "Additionally, chimeras of consensus sequences were removed"

> This should be explained in more detail.

3. Page 5, Line 110: "standard parameters"

> Should "standard" parameters be "default" parameters? If so, make that change. If not, list the parameters.

4. To make this work reproducible, all versions of all software and databases used in this study should be listed including (FALCON-Unzip, OrthoMCL, MCL, Gblocks, MAKER, PRANK, TimeTree, RAxML, PAML, Pfam, EggNOG, etc. There are others). Also all command lines should be included as a supplemental file. (See the docx file in the supplement of the following study for an excellent example of best practices in providing a detailed set of command lines:
https://academic.oup.com/mbe/article/35/2/486/4644721

5. Page 5, Line 113: "202 1:1:1 single-copy orthologous genes"

> It's confusing (and unnecessary) to label single-copy orthologs as "1:1:1 single-copy orthologs" when dealing with orthologs from 14 species. It would make sense with 3 species, but with 14 it would be 1:1:1:1:1:1:1:1:1:1:1:1:1:1, which would be a bit much.

6. Page 5, Line 115: "Gblock"

> Gblocks

7. Page 6, Line 130: "A statistical analysis of the changes in gene family sizes indicated significantly greater gene family expansion in O. minor (178 gene families) compared to other species"

> What is the statistical test? What is the P-value? What is considered significant (e.g. P <0.5)? How are gene families defined? Compared to which species? Does this mean that 178 gene families are expanded?

> Assemblies of PacBio sequence data (including those done by Falcon Unzip) suffer from the inclusion of multiple haplotigs per genomic locus. What tests have been done to be control for this? How do the authors

know that the expansion of gene families is not artifactual due to haplotigs?

Page 6, Line 148: "The larger gene size"
> I think the authors mean "larger number of genes." "Larger gene size" seems to refer to the number of nucleotides in genes.

Page 6, Line 142: "of repetitive sequences (44.43%)"—"Repeats accounted for 44%"

> Remove one of these 44%s --- It's repetitive.

Page 6, Line 142: "Repeats accounted for 44% (2.262 Gb) of the assembly, and were dominated by simple repeats (14.7%) and TEs"

> It's unclear whether 14.7% refers to the 14.7% of the genome or 14.7% of the repeats. Be explicit.

> Also, this paragraph would benefit by a side-by-side comparison of repeats and genes between the two Octopus. E.g. "O.minor genome is composed of 44% repeats and X% gene coding sequence, while O. maculoides genome consists of X% repeats and X% gene coding sequence." This could be helped by a table showing side-by-side values. As it is written it is difficult to get a feel for how the content of these genomes compare. I would also wait to talk about TEs, transposons, and LINEs until the next paragraph.

Page 6, Line 151: "TEs are crucial components"

> I would argue that since TEs are absent from some animal genomes, they are not "crucial." I suggest removing "crucial". Minor point.

BUSCO: Busco scores should be reported in the paper rather than in the FTP site. This should include: Total number of core genes queried, Number of core genes detected—Complete, Number of core genes detected—Complete + Partial, Number of missing core genes, Average number of orthologs per core genes, % of detected core genes that have more than 1 ortholog

Level of Interest

Please indicate how interesting you found the manuscript: Choose an item.

Quality of Written English

Please indicate the quality of language in the manuscript: Choose an item.

Declaration of Competing Interests

Please complete a declaration of competing interests, considering the following questions:

- Have you in the past five years received reimbursements, fees, funding, or salary from an organisation that may in any way gain or lose financially from the publication of this manuscript, either now or in the future?
- Do you hold any stocks or shares in an organisation that may in any way gain or lose financially from the publication of this manuscript, either now or in the future?
- Do you hold or are you currently applying for any patents relating to the content of the manuscript?

- Have you received reimbursements, fees, funding, or salary from an organization that holds or has applied for patents relating to the content of the manuscript?
- Do you have any other financial competing interests?
- Do you have any non-financial competing interests in relation to this paper?

If you can answer no to all of the above, write 'I declare that I have no competing interests' below. If your reply is yes to any, please give details below.

I declare that I have no competing interests.

I agree to the open peer review policy of the journal. I understand that my name will be included on my report to the authors and, if the manuscript is accepted for publication, my named report including any attachments I upload will be posted on the website along with the authors' responses. I agree for my report to be made available under an Open Access Creative Commons CC-BY license (http://creativecommons.org/licenses/by/4.0/). I understand that any comments which I do not wish to be included in my named report can be included as confidential comments to the editors, which will not be published.

I agree to the open peer review policy of the journal

To further support our reviewers, we have joined with Publons, where you can gain additional credit to further highlight your hard work (see: https://publons.com/journal/530/gigascience). On publication of this paper, your review will be automatically added to Publons, you can then choose whether or not to claim your Publons credit. I understand this statement. Yes
