# Supplementary material for: The genome of common long-arm octopus Octopus minor
Source: Gigascience. 2018 Sep 25;7(11):giy119. doi: 10.1093/gigascience/giy119 (PMC6279123; doi:10.1093/gigascience/giy119)
Supplement: Reviewer_2_Report_(Original_Submission) -- Yi-Jyun Luo — 6/24/2018 Reviewed [file giy119_reviewer_2_report_(original_submission).pdf]

Reviewer Report

Title: The genome of common long-arm octopus Octopus minor

Version: Original Submission **Date:** 6/24/2018

Reviewer name: Yi-Jyun Luo

Reviewer Comments to Author:

In the present manuscript, the authors provide the genome of the common long-arm octopus Octopus minor. It has been reported that the genome of the California two-spot octopus O. bimaculoides has a high amount of repeat content and several gene family expansions related to its morphological novelty. O. minor is closely related to O. bimaculoides, belonging to the same genus. The authors compared gene families and repetitive elements of these two octopus genomes with other lophotrochozoans and concluded that these two octopus genomes seem to be evolved independently.

Overall, this is a significant contribution to the field of cephalopod genomics. In order to support their hypothesis, the authors should address the issue of phylogenetic analyses of major gene families and repeats before publication.

Major comments:

1. The manuscript is well-written and straightforward. However, I find that there is a lack of evidence to show which events are related to Octopus genus-specific events or those of species-specific. Since one major conclusion from gene family and repeat analyses is that O. minor and O. bimaculoides evolved independently, the authors should provide evidence to test their hypothesis. For example, one major finding in the O. bimaculoides genome is that gene family expansions of protocadherins and the C2H2 superfamily of zinc-finger genes. Given that we have an additional genome from the same genus, the authors should provide gene trees to show that if these gene family expansions are general to the genus Octopus, or there was a convergent evolution in which these gene family expanded independently.

2. Also, it is worth to check the genomic organization of these gene family expansions in two octopus genomes. Are they usually expanded in a tandemly duplicated manner on the same scaffold? Or are they distributed among different scaffolds?

3. Similar situation for the repetitive elements, although the authors showed that the repeat landscape is different between two octopus genomes, there is no information about which repeat expansions have happened at the genus-level and which are at the species-level. The authors should at least examine some representative repetitive elements in details by providing their phylogenetic analysis with repeat trees.

4. In addition, the authors mentioned that they did RNA-seq of 13 tissues, but there is no description of this dataset. Are there some gene family expansions related to tissue-specific expression? The authors should provide some results from their RNA-seq data.

Minor comments:

1. Introduction: Given that octopuses are members of lophotrochozoans and the authors also used a lot of lophotrochozoan genomes for comparisons, the authors should properly describe previous work related to this topic. I would suggest the authors add some description about the relationship of molluscs and other lophotrochozoans and cite major papers to give an overview for the rationale of phylogenetic and gene analyses.

References:

Takeuchi et al. (2012) Draft genome of the pearl oyster Pinctada fucata: a platform for understanding bivalve biology. DNA Res 19, 117-30.

Zhang et al. (2012) The oyster genome reveals stress adaptation and complexity of shell formation. Nature 490, 49-54.

Simakov et al. (2013) Insights into bilaterian evolution from three spiralian genomes. Nature 493, 526-31.

Luo et al. (2015) The Lingula genome provides insights into brachiopod evolution and the origin of phosphate biomineralization. Nat Commun 6, 8301.

2. Line 12: "bilaterian animal species" -> "bilaterian species". Bilaterians are bilaterally symmetric animals, so using "bilaterian animal" would be redundant.

3. Line 40: Most O. minor habitats are "mud and sand"…

4. Line 42: The following sentence is unrelated to the scientific study, especially for the later part: "As an important economic cephalopod in South Korea, fishermen normally catch O. minor by digging a hole in the mudflat with shovels."

5. The Results section (or Analyses) "Genome sequencing and annotation" looks like for the Methods section. Should that be called "Data description" in GigaScience format?

6. Line 61: The authors should describe the strategy and sequencing platform they used. It is mentioned in the RNA part at line 73 but not for DNA. Did authors use the same strategy here?

7. Line 64: What kinds of paired-end sequences were used?

8. Line 69: thirteen -> "13".

9. Line 72: Remove "TM".

10. Line 73: Pacbio -> "PacBio".

11. Line 124: O. bimaculoides.

Level of Interest

Please indicate how interesting you found the manuscript: Choose an item.

Quality of Written English

Please indicate the quality of language in the manuscript: Choose an item.

Declaration of Competing Interests

Please complete a declaration of competing interests, considering the following questions:

- Have you in the past five years received reimbursements, fees, funding, or salary from an organisation that may in any way gain or lose financially from the publication of this manuscript, either now or in the future?

- Do you hold any stocks or shares in an organisation that may in any way gain or lose financially from the publication of this manuscript, either now or in the future?
- Do you hold or are you currently applying for any patents relating to the content of the manuscript?
- Have you received reimbursements, fees, funding, or salary from an organization that holds or has applied for patents relating to the content of the manuscript?
- Do you have any other financial competing interests?
- Do you have any non-financial competing interests in relation to this paper?

If you can answer no to all of the above, write 'I declare that I have no competing interests' below. If your reply is yes to any, please give details below.

I declare that I have no competing interests.

I agree to the open peer review policy of the journal. I understand that my name will be included on my report to the authors and, if the manuscript is accepted for publication, my named report including any attachments I upload will be posted on the website along with the authors' responses. I agree for my report to be made available under an Open Access Creative Commons CC-BY license (http://creativecommons.org/licenses/by/4.0/). I understand that any comments which I do not wish to be included in my named report can be included as confidential comments to the editors, which will not be published.

I agree to the open peer review policy of the journal

To further support our reviewers, we have joined with Publons, where you can gain additional credit to further highlight your hard work (see: https://publons.com/journal/530/gigascience). On publication of this paper, your review will be automatically added to Publons, you can then choose whether or not to claim your Publons credit. I understand this statement. Yes
