# Supplementary material for: The genome of common long-arm octopus Octopus minor
Source: Gigascience. 2018 Sep 25;7(11):giy119. doi: 10.1093/gigascience/giy119 (PMC6279123; doi:10.1093/gigascience/giy119)
Supplement: Response_To_Reviewer_Comments_Revision_1.pdf [file giy119_response_to_reviewer_comments_revision_1.pdf]

Author's Response To Reviewer Comments

Close

Your manuscript "The genome of common long-arm octopus Octopus minor" (GIGA-D-18-00174R1) has been assessed by our reviewers. Based on these reports, and my own assessment as Editor, I am pleased to inform you that it is potentially acceptable for publication in GigaScience, once you have carried out some essential revisions suggested by our reviewers.
Response: Thank you so much for your work. We have corrected it as reviewer comments.

Reviewer #1: The authors addressed all of my concerns.
Response: Thank you so much for your all of review.

Reviewer #2: In general, the authors ignored my comments and addressed that there is a need to reduce the analysis part for a Data Note. I can understand this consideration if that is an editorial policy of the journal. I have no further comments but some corrections for typos.
Page 6, Line 122 & Page 9, Line 234-236: CAFE is the acronym for "Computational Analysis of gene Family Evolution", but not "coffee" in French. Please replace the uppercase "E-acute" with the ordinary uppercase "E". Same for Table S6-S8.
Response: Thank you so much for your all of review. We have corrected it.

Page 7, Line 159: The acronym "TE" suddenly appears without being spelled out in the main text.
Response: We have corrected it. We wrote full name in first time.

Close
