# Supplementary material for: The genome of common long-arm octopus Octopus minor
Source: Gigascience. 2018 Sep 25;7(11):giy119. doi: 10.1093/gigascience/giy119 (PMC6279123; doi:10.1093/gigascience/giy119)
Supplement: Response_To_Reviewer_Comments_(Original_Submission).pdf [file giy119_response_to_reviewer_comments_(original_submission).pdf]

Author's Response To Reviewer Comments

Close

From an editorial perspective we also have some requirements. Please include a better picture of the species, and also for QC/validation purposes please include a basic phylogenetic tree (e.g. comparisons with other sequenced Cephalopoda). We also require a statement that you followed ethical norms and had animal research board approval. The points the reviewers have raised regarding reproducibility and data access are very important, so make sure you include all accession numbers, software details (or copy data and custom scripts to GigaDB), and RRIDs:

Response: We add photo of species in Fig 1a. Phylogenetic tree with other sequenced Cephalopoda and mollusks was included in Fig. 2a. And ethic statement, accession numbers and software details version are included in manuscript.

Reviewer reports:

Reviewer #1: This is a nicely written data note describing a very interesting and important genomic resource, the genome of Octopus minor. The data and assembly seem reasonable.

1. Page 3, Line 24: "As advanced invertebrates,"
> "Advanced" implies that these animals have been evolving longer than other invertebrates. This is not true and this sentence would be improved if this phrase was removed.

Response: As the reviewer suggested, we have corrected the sentence.

2. Page 4, Line 78: "Additionally, chimeras of consensus sequences were removed"
> This should be explained in more detail.

Response: HQ isoform data generated using TOFU pipeline exists in the form of a chimera-like PCR Chimera. Therefore, an additional removal process is required. This part was removed using the in-house script. We have provided the script in supplementary text.

3. Page 5, Line 110: "standard parameters"
> Should "standard" parameters be "default" parameters? If so, make that change. If not, list the parameters.

Response: As the reviewer suggested, we have corrected the sentence.

4. To make this work reproducible, all versions of all software and databases used in this study should be listed including (FALCON-Unzip, OrthoMCL, MCL, Gblocks, MAKER, PRANK, TimeTree, RAxML, PAML, Pfam, EggNOG, etc. There are others). Also all command lines should be included as a supplemental file. (See the docx file in the supplement of the following study for an excellent example of best practices in providing a detailed set of command lines:

https://academic.oup.com/mbe/article/35/2/486/4644721

Response: Thank you so much for your valuable suggestions, we have made a extra supplementary note describing all the commands used for genome analysis processes.

5. Page 5, Line 113: "202 1:1:1 single-copy orthologous genes"
> It's confusing (and unnecessary) to label single-copy orthologs as "1:1:1 single-copy orthologs" when dealing with orthologs from 14 species. It would make sense with 3 species, but with 14 it would be 1:1:1:1:1:1:1:1:1:1:1:1:1:1, which would be a bit much.

Response: As the reviewer suggested, we have corrected the sentence.

6. Page 5, Line 115: "Gblock"
> Gblocks

Response: As the reviewer suggested, we have corrected the sentence.

7. Page 6, Line 130: "A statistical analysis of the changes in gene family sizes indicated significantly greater gene family expansion in O. minor (178 gene families) compared to other species"
> What is the statistical test? What is the P-value? What is considered significant (e.g. P <0.5)? How are gene families defined? Compared to which species? Does this mean that 178 gene families are expanded?

Response: Sorry for the confusion. All the results are describing about gene loss-gain analysis. To make it clear, we have corrected the sentence and have added p-value cut off used for CAFÉ analysis.

> Assemblies of PacBio sequence data (including those done by Falcon Unzip) suffer from the inclusion of multiple haplotigs per genomic locus. What tests have been done to be control for this? How do the authors know that the expansion of gene families is not artifactual due to haplotigs?

Response: We performed gene family analysis using only the primary assembly in assembly results generated by Falcon-unzip. Therefore, we do not expect any analysis error due to haplotigs interference.

Page 6, Line 148: "The larger gene size"
> I think the authors mean "larger number of genes." "Larger gene size" seems to refer to the number of nucleotides in genes.

Response: As the reviewer suggested, we have corrected the sentence.

Page 6, Line 142: "of repetitive sequences (44.43%)"—"Repeats accounted for 44%"
> Remove one of these 44%s --- It's repetitive.

Response: As the reviewer suggested, we have removed that part.

Page 6, Line 142: "Repeats accounted for 44% (2.262 Gb) of the assembly, and were dominated by simple repeats (14.7%) and TEs"

> It's unclear whether 14.7% refers to the 14.7% of the genome or 14.7% of the repeats. Be explicit.
> Also, this paragraph would benefit by a side-by-side comparison of repeats and genes between the two Octopus. E.g. "O.minor genome is composed of 44% repeats and X% gene coding sequence, while O. maculoides genome consists of X% repeats and X% gene coding sequence." This could be helped by a table showing side-by-side values. As it is written it is difficult to get a feel for how the content of these genomes compare. I would also wait to talk about TEs, transposons, and LINEs until the next paragraph.

Response: We are sorry for not organized sentences. As the reviewer suggested, we have made clear the sentences describing brief differences of genome characteristics between O. minor and O. bimaculoides.

Page 6, Line 151: "TEs are crucial components"
> I would argue that since TEs are absent from some animal genomes, they are not "crucial." I suggest removing "crucial". Minor point.

Response: As the reviewer suggested, we have corrected the sentence.

BUSCO: Busco scores should be reported in the paper rather than in the FTP site. This should include: Total number of core genes queried, Number of core genes detected—Complete, Number of core genes detected—Complete + Partial, Number of missing core genes, Average number of orthologs per core genes, % of detected core genes that have more than 1 ortholog

Response: Thank you for your suggestions. We have moved the supplementary table 2 describing BUSCO results to main table 1.

Reviewer #2: In the present manuscript, the authors provide the genome of the common long-arm octopus Octopus minor. It has been reported that the genome of the California two-spot octopus O. bimaculoides has a high amount of repeat content and several gene family expansions related to its morphological novelty. O. minor is closely related to O. bimaculoides, belonging to the same genus. The authors compared gene families and repetitive elements of these two octopus genomes with other lophotrochozoans and concluded that these two octopus genomes seem to be evolved independently.

Overall, this is a significant contribution to the field of cephalopod genomics. In order to support their hypothesis, the authors should address the issue of phylogenetic analyses of major gene families and repeats before publication.

Major comments:

1. The manuscript is well-written and straightforward. However, I find that there is a lack of evidence to show which events are related to Octopus genus-specific events or those of species-specific. Since one major conclusion from gene family and repeat analyses is that O. minor and O. bimaculoides evolved independently, the authors should provide evidence to test their hypothesis. For example, one major finding in the O. bimaculoides genome is that gene family expansions of protocadherins and the C2H2 superfamily of zinc-finger genes. Given that we have an additional genome from the same genus, the authors should provide gene trees to show that if these gene family expansions are general to the genus Octopus, or there was a convergent evolution in which these gene family expanded independently.

Response: Thank you for the positive comment on our manuscript. Based on the reviewer’s comment, we analyzed genomic expansions of protocadherins and C2H2 zinc finger gene family from the O. minor genome. In the case of squid, there is no genome information available yet. However, from the transcriptome data, only small numbers of protocadherins and C2H2 zinc finger gene family were identified in squid (Albertin et al., 2015). Moreover, Albertin et al. (2015) measured that octopus protocadherins appear to have expanded ~135 Mya after octopuses diverged from squid. In our study, we estimated that O. minor was diverged from O. bimaculoides. Thus, we assume that the extraordinary expansions of both gene families are Octopus-specific. Sentences incorporated in the revised manuscript are appended as follows;

Previously, 168 protocadherin (pcdhs) genes were annotated in the genome of O. bimaculoides, which is the largest number among sequenced metazoan genomes (Figure S8.3.2 in Albertin et al., 2015). In the case of C2H2 zinc finger gene family, approximately 1,800 C2H2 genes were annotated in the O. bimaculoides genome. The drastic expansions were also observed in the genome of O. minor, as 303 and 2,289 genes were annotated for pcdhs and C2H2 zinc finger gene family, respectively. We assume that the expansion patterns are unique to the genus Octopus, as the expansion pattern was not detected in squid and the pcdhs seem to have expanded after octopuses diverged from squid (≈ 135 Mya) (Albertin et al., 2015). Since we estimated that O. minor diverged from the genus Octopus, the extraordinary expansions of both gene families are presumably Octopus-specific.

2. Also, it is worth to check the genomic organization of these gene family expansions in two octopus genomes. Are they usually expanded in a tandemly duplicated manner on the same scaffold? Or are they distributed among different scaffolds?

Response: Thank you so much for your informative comments. Unfortunately, we have needed to reduce biological analysis part to follow data note author guidelines. Following your valuable suggestions, we are going to analysis gene family organizations in our future study.

3. Similar situation for the repetitive elements, although the authors showed that the repeat landscape is different between two octopus genomes, there is no information about which repeat expansions have happened at the genus-level and which are at the species-level. The authors should at least examine some representative repetitive elements in details by providing their phylogenetic analysis with repeat trees.

Response: Similar with the previous response, we had to reduced the analysis part. Thank you so much for your suggestions.

4. In addition, the authors mentioned that they did RNA-seq of 13 tissues, but there is no description of this dataset. Are there some gene family expansions related to tissue-specific expression? The authors should provide some results from their RNA-seq data.

Response: Like previous response, we had to reduce the biological analysis part. In this manuscript, we have used RNA-seq data to annotate genes. We are going to analysis tissue-specific RNA expression patterns in the near future.

Minor comments:

1. Introduction: Given that octopuses are members of lophotrochozoans and the authors also used a lot of lophotrochozoan genomes for comparisons, the authors should properly describe previous work related to this topic. I would suggest the authors add some description about the relationship of molluscs and other lophotrochozoans and cite major papers to give an overview for the rationale of phylogenetic and gene analyses.
References:
Takeuchi et al. (2012) Draft genome of the pearl oyster Pinctada fucata: a platform for understanding bivalve biology. DNA Res 19, 117-30.
Zhang et al. (2012) The oyster genome reveals stress adaptation and complexity of shell formation. Nature 490, 49-54.
Simakov et al. (2013) Insights into bilaterian evolution from three spiralian genomes. Nature 493, 526-31.
Luo et al. (2015) The Lingula genome provides insights into brachiopod evolution and the origin of phosphate biomineralization. Nat Commun 6, 8301.

Response: Thank you so much for your suggestion and references. We have added introductory sentence about genome information scarcity of mollusk and their relationship with lophotrochozoans.

2. Line 12: "bilaterian animal species" -> "bilaterian species". Bilaterians are bilaterally symmetric animals, so using "bilaterian animal" would be redundant.

Response: As the reviewer suggested, we have corrected the sentence.

3. Line 40: Most O. minor habitats are "mud and sand"…

Response: As the reviewer suggested, we have corrected the sentence.

4. Line 42: The following sentence is unrelated to the scientific study, especially for the later part: "As an important economic cephalopod in South Korea, fishermen normally catch O. minor by digging a hole in the mudflat with shovels."

Response: As the reviewer suggested, we have corrected the sentence.

5. The Results section (or Analyses) "Genome sequencing and annotation" looks like for the Methods section. Should that be called "Data description" in GigaScience format?

Response: As the reviewer suggested, we have corrected the sentence.

6. Line 61: The authors should describe the strategy and sequencing platform they used. It is mentioned in the RNA part at line 73 but not for DNA. Did authors use the same strategy here?

Response: As the reviewer suggested, we have corrected the sentence.

7. Line 64: What kinds of paired-end sequences were used?

Response: As the reviewer suggested, we have corrected the sentence.

8. Line 69: thirteen -> "13".

Response: As the reviewer suggested, we have corrected the sentence.

9. Line 72: Remove "TM".

Response: As the reviewer suggested, we have corrected the sentence.

10. Line 73: Pacbio -> "PacBio".

Response: As the reviewer suggested, we have corrected the sentence.

11. Line 124: O. bimaculoides.

Response: As the reviewer suggested, we have corrected the sentence.

Close
